# Professional or Amateur? The Phonological Output Buffer as a Working Memory Operator

**DOI:** 10.3390/e22060662

**Published:** 2020-06-15

**Authors:** Neta Haluts, Massimiliano Trippa, Naama Friedmann, Alessandro Treves

**Affiliations:** 1 Language and Brain Lab, Sagol School of Neuroscience and School of Education, Tel Aviv University, Tel Aviv-Yafo 69978, Israel; nhaluts@gmail.com (N.H.); naamafr@tauex.tau.ac.il (N.F.); 2 SISSA—Cognitive Neuroscience, Via Bonomea 265, 34136 Trieste, Italy; max.trippa@gmail.com

**Keywords:** phonological output buffer, Potts network, cortex, latching dynamics, working memory

## Abstract

The Phonological Output Buffer (POB) is thought to be the stage in language production where phonemes are held in working memory and assembled into words. The neural implementation of the POB remains unclear despite a wealth of phenomenological data. Individuals with POB impairment make phonological errors when they produce words and non-words, including phoneme omissions, insertions, transpositions, substitutions and perseverations. Errors can apply to different kinds and sizes of units, such as phonemes, number words, morphological affixes, and function words, and evidence from POB impairments suggests that units tend to substituted with units of the same kind—e.g., numbers with numbers and whole morphological affixes with other affixes. This suggests that different units are processed and stored in the POB in the same stage, but perhaps separately in different mini-stores. Further, similar impairments can affect the buffer used to produce Sign Language, which raises the question of whether it is instantiated in a distinct device with the same design. However, what appear as separate buffers may be distinct regions in the activity space of a single extended POB network, connected with a lexicon network. The self-consistency of this idea can be assessed by studying an autoassociative Potts network, as a model of memory storage distributed over several cortical areas, and testing whether the network can represent both units of word and signs, reflecting the types and patterns of errors made by individuals with POB impairment.

## 1. The Phonological Output Buffer and Its Challenges

The phonological output buffer (POB) is a cognitive component that is usually regarded as having a twofold function: first, it is a phonological working memory (pWM) component responsible for maintaining the phonological information of a word until it has been articulated [1,2,3]. Second, the POB is responsible for the composition of words from smaller units, as it assembles separate phonemes into words, and stems and affixes into morphologically complex words [4,5,6]. Thus, the POB is involved in the late production stages of word retrieval, reading and repetition of words and non-words. In processes involving existing words, such as naming, the POB receives input from a long-term store called the Phonological Output Lexicon (POL), which stores the phonological information of familiar words. However, in processes involving non-words, the POB is not supported by the POL, but rather receives input from phoneme-level processes, such as grapheme-to-phoneme conversion in non-word reading [5,7].

How does it work? To write a short code that implements the maintenance and assembly functions of the POB seems rather trivial. However, the human cortex does not write codes, and it does not appear to be endowed with circuitry intended to serve specifically as a POB. How a remarkably efficient POB can self-organize from standard cortical components—those shared with other mammals—remains rather mysterious. A possible approach to address this issue is to start from POB malfunction, i.e., to combine an analysis of its successful performance with that of its occasional failings, as observed more frequently in certain patients. Our goal here is not to develop a connectionist model, but rather a minimalistic first sketch of how the POB could emerge from ordinary neural resources, focusing on its main points of failure, in order to understand the critical implementational constraints that shape its computation. The complexity of the brain, in which it is not entirely clear what the ’standard cortical components’ available by default are in the mammalian cortex or, say, specifically in human BA 44 or 45 [8], make even this modest first sketch rather non-trivial.

Patients with developmental or acquired impairments to the POB (output conduction aphasia) tend to make phonological errors in repeating, reading aloud and producing words and non-words—such as omissions, insertions, transpositions, substitutions of one phoneme with another, as well as perseverations of phonemes from previous words or from the same word (e.g., substituting the phoneme /r/ with the phoneme /z/, resulting in production of ‘zebza’ instead of ‘zebra’, or transposing the phoneme /r/ and the phoneme /b/ resulting in the production of ‘zerba’) [5,9,10,11]. The lexical representation in the POL may support the POB in production, repetition, and reading of existing words, but not non-words, which are not stored in any lexicon. Thus, the POB is affected by a lexicality effect: existing words are easier for POB-impaired patients to produce, repeat, and read aloud than non-words due to the lexical support from the POL to the POB [5,6,12,13]. Still, patients with POB impairment also make errors on words, mainly on longer, morphologically complex words, or when they need to recall several words together, in span tasks for example.

Neuropsychological evidence has revealed that in addition to the phoneme-level errors that these patients make, they also make whole-unit errors in morphological affixes, function words, and number words. This means that, instead of errors involving single phonemes within the unit, the errors involve the unit as a whole (e.g., producing the number word ‘eight’ instead of ‘five’). These whole-unit errors include omissions and insertions of the whole affix, function word, or number word, and critically—they involve substitutions of one unit with another one of the same category: affixes with other affixes, function words with other function words [5,9,10], and number words with other number words [14,15,16,17]. This pattern of errors leads to sensitivity to the morphological status of the word to-be-articulated—when repeating or producing morphologically complex words and non-words, POB impaired patients make phonological errors in the stem of the word, and whole-unit errors in the morphological affix. Thus, cases of POB impairments reveal that phonemes are not the only type of unit used by the POB: phonemes, morphological affixes, function, and number words all act as units that are composed together and held by the POB. Therefore, the second function of the POB can be described more generally: the POB is responsible for the composition of words and phrases from smaller units—which are either phonemes, in the case of morphologically simple words and non-words, and stems of morphologically complex words, or whole affixes, function and number words in cases where the production of this type of units is required. In the case of spoken languages, these units are (mostly) sequential (e.g., phonemes are produced one after the other, rather than occurring simultaneously); however, as we explain in the following section, units can also occur simultaneously, as is most prominent in sign languages (but also in some processes in spoken languages, as in the case of roots and patterns in Semitic languages [18,19]).

Importantly, the phonological form itself is not the one that determines whether a phonological or a whole-unit error takes place: when the same phonological form (e.g., of a number word) appears in a non-numeric context, the errors within this form would be phonological. Only when the same form appears in a numeric context as a number word, the errors would be within-category errors [5]. This pattern of errors thus supports the building blocks hypothesis [14], according to which units that act as building blocks in a productive process are stored as pre-assembled units of some sort in separate mini-stores within the POB.

From a purely computational point of view, e.g., that taken by connectionist models, a unit can be pre-assembled by assigning it a new variable in a computer code, which can then be fed into the routine, perhaps dressed up as a neural network, which produces the composition or higher order assembly assigned computationally to the POB. From a neural point of view, however, it is unclear what allocating a new variable could mean: the cortex does not define new variables with a click on a key; unless it means the (laborious) formation of a new cell assembly, i.e., gradually endowing a distributed representation, over neurons that are already there and that individually participate in the representation of many other items, with the attractor properties that enable it to operate as a quasi-discrete unit [20]. A serial composition of building blocks would then be realized as a non-trivial, error prone concatenation of attractor states. A simultaneous composition, on the other hand, implies the concurrent activation of several attractor states, which might be relatively straightforward if the corresponding distributed representations were physically separated on distinct patches of cortex, but is quite challenging when they overlap on the same neuronal populations.

Because POB-impaired patients make within-category errors, i.e., they substitute units with other units of the same type (e.g., one number word with another), but do not make between categoeries errors (e.g., they do not substitute a number with a morphological affix; number words and morphemes do not “break” into phonemes, and there are no sub-morpheme or sub-number word errors) one should assume that the cell assemblies representing building blocks of the same type, e.g., morphemes, are distributed over the same population of neurons, whereas the evidence is inconclusive regarding those that represent building blocks of different types: they may not be confused because they are physically separated on different substrates, or because their attractor states are effectively segregated dynamically—even though they can be concatenated, as when a morpheme follows a series of phonemes. There is obviously a need for a concrete model that allows for assessing these putative dynamics explicitly, through simulations and, if possible, mathematical analysis.

Another important effect stemming from the WM function of the POB is a per-phoneme length effect—as shown in many studies about the pWM capacity for repeating lists of long words compared to lists of short words, e.g., [21,22]. It was discovered that, in POB patients, even single long words are harder to say, repeat and read aloud than short words, so that the probability of making a phonological error increases with word length [23,24,25], i.e., long words lead to a greater per-phoneme error rate than short words [5]. This is considered to stem from limited WM capacity, according to some studies due to sensitivity to the number of phonemes that have to be concurrently retained or to phonological complexity, and according to other studies due to sensitivity to the temporal duration of this temporary retention, or the time needed for them to be articulated or rehearsed in the pWM [21,22,26,27,28,29,30,31,32,33]. The suggestion, in either case, is that the POB is not operating on neural representations created *ad hoc*, or using special neural mechanisms qualitatively different from other manifestations of WM in cognitive processes. Therefore, in this respect, the POB provides a window into the limits and capabilities of WM as implemented in cortical circuitry. A most useful window, given the rich set of phenomena that can be observed in naturalistic settings, in people with normal POB and in those with specific impairments to the pWM. To begin to understand the computational challenges underlying this rich phenomenology, we propose to focus first on a comparison between spoken and sign languages.

## 2. Sign Language Phonology and Its Distinct Demands on an Output Buffer

In spoken languages, phonemes are the sound elements under the level of the word and morpheme. Phonemes have no meaning of their own but are the minimal contrastive units within a language creating meaning differences between words. Replacing a phoneme within a word with another can create a difference in meaning. Phonemes are comprised of phonological features (such as place of articulation, manner of articulation, and voicing), and the distinctive features within a language create differences between phonemes. Pairs of words that have different meanings and differ in only one phonological feature are called ‘minimal pairs’ (e.g., /r/ and /l/ are two separate phonemes in English, as shown by the fact that they create pair of words that differ in meaning: *road* and *load*, for example).

**Figure 1:**
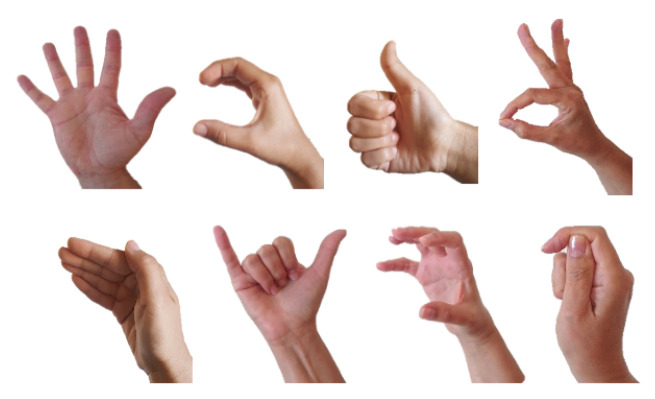
Examples of different handshapes in Israeli Sign Language (ISL).

**Figure 2:**
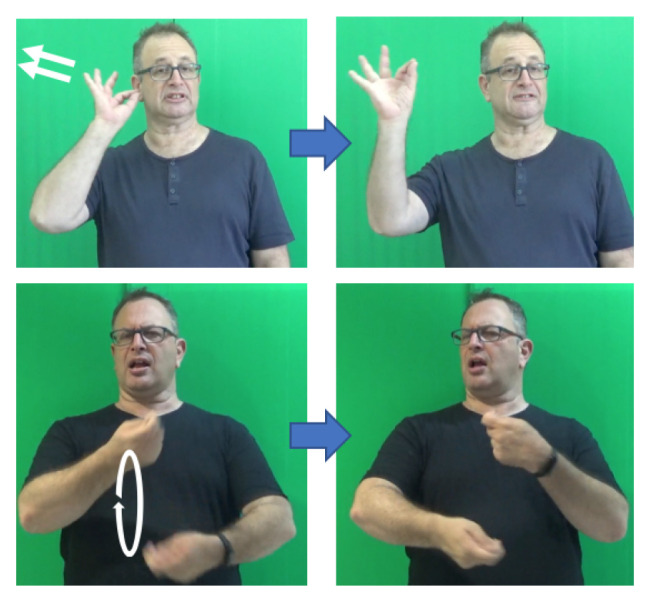
Examples of different movements in ISL signs. Top—the sign BEEP signed with a straight repeated movement, bottom—the sign ECONOMY signed with a circular movement.

**Figure 3:**
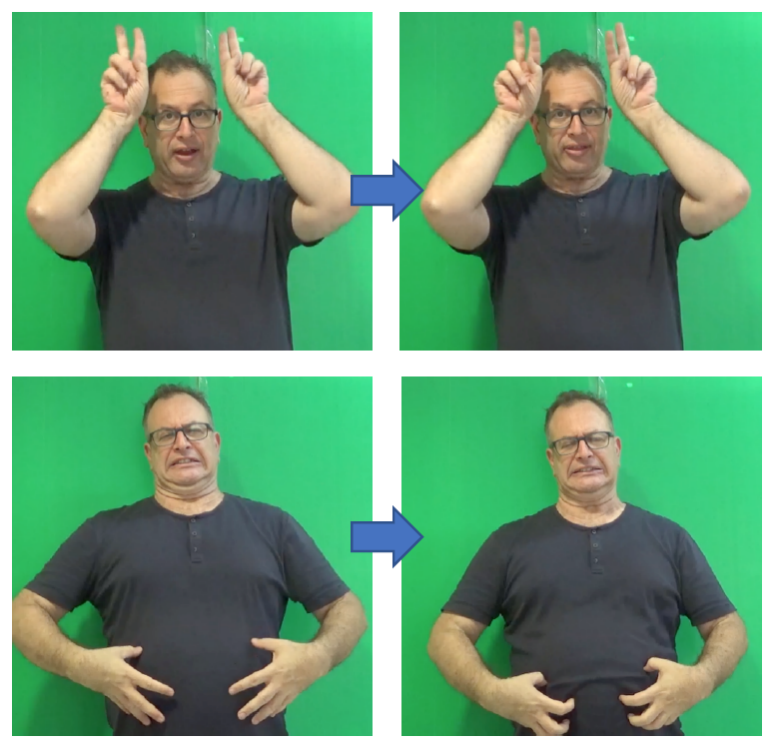
Examples of different locations in ISL signs. Top—the sign BUNNY signed on top of the head, bottom—the sign CONTRACTION signed on the stomach.

The notion of phonology is concerned with the patterns of phonemes, their analysis in terms of phonological features, and their form and organization within a language [34]. However, it is not unique to spoken languages and sounds. Already in the 1960’s, it was noted that sign languages also exhibit a structural level parallel to phonology [35,36,37,38]. Stokoe [35] has shown that signs are comprised of a finite set of linguistically significant meaningless elements. The three main categories of contrastive elements in sign languages are handshape, movement, and location (or place of articulation), since they act as distinctive elements in minimal pairs of signs. Figure 1, Figure 2, and Figure 3 show examples of possible handshapes, movements, and locations, respectively, in Israeli Sign Language (ISL). Later models have suggested that these three main parameters (which we will refer to as “sign-elements”) are comprised of combinations of, or hierarchically organized, phonological features (note that non-manual elements, such as facial expressions, may also co-occur with the lexical sign, acting as phonological elements; however, this type of non-manual features will not be considered here). A major difference between the organization of phonemes in spoken languages and sign languages is the balance between simultaneous and sequential realization. Phonemes within a spoken word are sequential—i.e., follow one another temporally; while it seems that the three sign-elements are realized largely simultaneously, without a temporal difference [35]. Nevertheless, whereas early models related to representation of signs as either completely simultaneous [35] or completely sequential [39], more recent models represent the phonology of signs as a combination of simultaneous and sequential features, with at least one element represented as a sequence of temporal units and the features of the other sign-elements spread across these units [37,38,40,41].

Therefore, it is important to consider these different levels of simultaneity in the phonological representation of words and signs when trying to account for shared mechanisms dealing with material of both types. Thus, a model of the POB that aims to account for both spoken and signed stimuli should be able to represent both sequential and simultaneous units.

### 2.1. Markedness of Handshapes

Markedness refers to relations between elements in phonological classes, and it captures the observation that not all phonological elements have the same status [42]. Unmarked elements are structurally simpler than marked elements, they allow a greater number of subtypes than more marked elements, i.e., they can surface in a larger number of variants, and they are more widely distributed than marked elements [43,44,45,46,47,48]. Based on these criteria, Brentari et al. [37] suggest some differences between marked and unmarked handshapes in sign languages, including:

1. Unmarked shapes are acquired earlier.
2. Unmarked shapes are easier to articulate.
3. Unmarked shapes can be used on H2 (the non-dominant hand), while marked shapes cannot.
4. Unmarked shapes are more frequent cross-linguistically.

**Figure 4:**
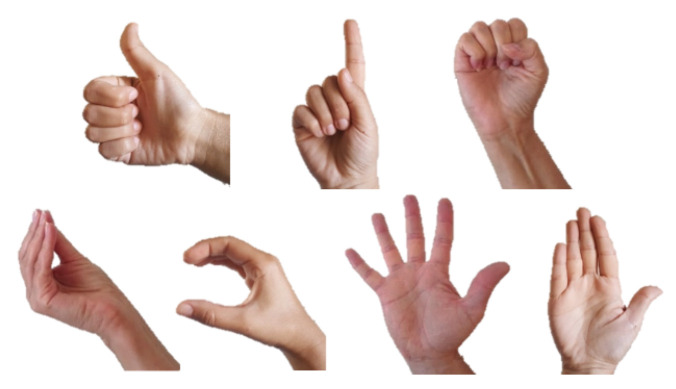
Unmarked handshapes in ISL, based on the American Sign Language (ASL) handshapes suggested in Brentari [53].

Brentari [37] show that the handshapes that can appear on H2 in American Sign Language (ASL) are indeed the less complex handshapes, because they require fewer specifications for the joints’ configuration and selected fingers. This means that the active, or “selected fingers”, which form the shape in unmarked handshapes are either all fingers, in a range of joint configurations, or only the index finger, which is extended. Furthermore, these handshapes have the widest distribution across ASL lexicon and have the widest range of subtypes. As to sensitivity to markedness, it seems to be the case that *marked* handshapes are more susceptible to errors in language acquisition and in aphasia than unmarked shapes [49,50,51,52]. A set of unmarked handshapes in ISL, modified based on the set of ASL unmarked shapes suggested in Brentari [53], can be seen in Figure 4. Haluts and Friedmann [54,55] show that the markedness of handshapes plays a role in the pWM of deaf signers: in repetition of lists of *non-signs* (parallel to non-words), marked handshapes were more frequently substituted and omitted than unmarked handshapes. This indicates that the need to recall marked handshapes places a greater load on the POB of signers than that of unmarked handshapes, causing more difficulty in memorizing the marked handshape and more errors with this type of handshapes. However, when a handshape was substituted, there was no preference of substituting it with an unmarked handshape. This means that unmarked handshapes, though easier for the phonological systems to memorize, are not the default form in production. Although it is far from clear what might differentiate different degrees of markedness at the neural level, one can conclude from this brief discussion that handshapes are unlikely to be associated simply with separate variables, as is often assumed in information-processing models. Any attempt to understand patterns of errors in their production should start from the inherent fragility of their neural codes.

### 2.2. The Non-Dominant Hand

One of the major phonetic differences between spoken and sign languages is that, in contrast to spoken languages, in the production of a sign language there are two identical articulators—the two hands, which can articulate simultaneously. This enables different types of signs, either one-handed or two-handed. However, it seems not to be the case that the two hands act as two independent articulators—whereas the dominant hand (H1) is relatively free regarding the handshapes and movements it can perform within a sign (out of the available phonemes within a language), the non-dominant hand (H2) exhibits a very restricted behaviour, and it is much more limited concerning the handshapes and movement types it can exhibit independently [37,38]. Two-handed signs can be divided into balanced signs, where the two hands are active (e.g., they both move) and must share a handshape, and unbalanced signs, where only the dominant hand is active [56,57]. The second group can be further divided based on the handshape exhibited by the non-dominant hand, yielding the three types of two-handed signs suggested by Battison (1978) (excluding a fourth type of a combination found in compound signs):

1. type-1: both hands are active, have the same handshape, and perform the same movement.
2. type-2: H2 is passive, and exhibits the same handshape as the dominant hand.
3. type-3: H2 is passive, but exhibits a different handshape from the dominant hand.

It seems, then, that, although the two hands can act as two independent articulators in theory, in fact, two-handed signs are very restricted regarding the role of the non-dominant hand. Thus, the phonological representations of one-handed and two-handed signs might not be very different.

Haluts [54] tested how the number of hands in a sign affects the POB, and found no significant difference between the percentage of one-handed lists and two-handed lists repeated correctly by each participant. Thus, it seems that despite the involvement of a second articulator—two-handed signs do not put a greater load on the pWM systems of signers. This example shows that phonetic differences do not necessarily indicate major differences in phonological representation. Thus, despite the phonetic difference between spoken and sign languages, their phonological representations might be more similar than it looks, and it might be that the same phonological mechanisms can deal with information of both modalities.

### 2.3. The Inventory of Phonemes in ISL

The inventory of phonemes—the contrastive elements in the language—changes from one language to another. Modern Hebrew, for example, has about 24 distinctive consonants (a number that varies depending on the specific tradition of pronunciation, and the status of phonemes in borrowed words), and five vowels [58,59]. Standard Italian has a similar number of consonants, and seven vowels [60]. An exhaustive inventory of phonemes in ISL was not published to date; an assessment can be drawn, however, from the published phonological findings. Meir and Sandler [61] present a list of the handshapes in ISL, which contains 25 distinctive handshapes and four handshapes borrowed from the fingerspelling alphabet. They also relate to five major locations in ISL—the signer’s head, trunk, non-dominant hand, arm, and the neutral space in front of the signer. These locations are further specified for features like height in respect to the place of articulation (high, medium, or low) and laterality with respect to the dominant hand (ipsi-lateral, contra-lateral, or central). Additional features (contact, proximal, and distal) are presented in Sandler and Lillo-Martin [38], but it is unclear whether all of them are distinctive in ISL. Thus, the theoretically possible locations in ISL are many, but there are probably considerably fewer distinctive locations that act as contrastive elements in ISL signs. The number of distinctive movements within a sign language seems to be even more complex to define, and different types of distinctive features have been suggested to account for the possible inventories. Sandler [62,63] considers arc, contact, tense, and restrained (or doubled) as distinctive features of lexical path movement in sign languages (and in ISL in particular). It seems that at least some of these features (e.g., arc and restrained) can be combined together in the same movement. Internal movements are represented according to this suggestion by branching of the handshape (or hand configuration) parameter (e.g., a change from a closed position of the fingers to an open position). For ASL, Friedman [64] has suggested an inventory of 29 handshape phonemes, four movement aspects (each containing 5–7 options for specific features) and four major areas, which are further divided into a set of about 17 distinctive locations. Liddell and Johnson [39] give a list of 24 major handshapes, 18 major body locations, and four groups of features describing movements, each containing 3–7 features. Although the different models vary regarding the number of distinctive handshapes and locations, and are not definite about distinctive movements, it appears that the number of contrastive elements for a given sign language is finite, but probably greater than that for an average spoken language. Clearly, a quantitative appraisal of sign variability would also require analyses of the possible combinations of elements, and of their relative frequency. Waiting for such measures to be taken, it already appears evident that the average entropy per sign should be considerably larger than the entropy per phoneme of a spoken language, and much closer to the average entropy per word—the main reason being the limited number of signs that are associated with a word, typically just one. A feature that requires a discussion of syllables.

### 2.4. The Syllable in Sign Languages

The simultaneous nature of the phonology in sign languages makes it harder to imagine how syllables can exist within a sign. However, some researchers claim that sign syllables do exist, and each syllable is formed around movement—which is claimed to be the most salient element within the sign (Chinchor, 1978 in [65]) [46,66,67,68], just like spoken language syllables are organized around a vowel. The movement parameter is sometimes regarded as parallel to the nucleus of a syllable, acting as its sonority peak. It seems that a well-formed sign must include some type of movement, even though movement, in contrast to handshape and location, is not obligatory from a phonetic point of view [62]. This movement can be path movement—of the hand from one location to another, or internal movement (or local movement)—a change in the orientation of the hand or the position of the fingers [62]. A combination of path and internal movement can occur within one sign, but one type of movement is preferred in most sign languages [63]. Brentari [37] defines a criterion for counting the number of syllables in a sign: the number of sequential phonological dynamic units in a string equals the number of syllables in that string. Note that according to this definition, movements that are signed concurrently (e.g., a movement of the arm and of the finger joints executed at the same time) are still considered one syllable, and a sign would be considered disyllabic only when movements appear one after the other. Since most signs across sign languages do not have more than one sequential dynamic unit, most lexical items in sign languages exhibit a mono-syllabic structure. Thus, if cognitive mechanisms are sensitive to the number of syllables within an item, many simple lexical signs would be compatible with mono-syllabic words. Thus, the structure of these mono-syllabic signs is relatively fixed, because they always contain the three main sign-elements.

Nevertheless, there are types of signs that tend to be disyllabic. One phenomenon that is widespread across sign languages, resulting in disyllabic signs is compounding. Compounds are single signs formed by concatenation of two existing lexical signs [69,70,71]. In regular compounds one sign acts as the head of the compound, and the other as its modifier, e.g., the concatenation of Israeli Sign Language (ISL) signs STUDY and UNIVERSITY results in the compound STUDYÛNIVERSITY, which means ‘student’, and in which STUDY is the head and UNIVERSITY is the modifier. Because the process requires concatenation of two (usually) mono-syllabic signs, the compound contains two sequential movements and is thus disyllabic. As part of the lexicalization of the new sign, the compound undergoes morpho-phonological changes in order to fit the phonological form of lexical signs within the sign language. These morpho-phonological chang es include:

1. Reduction of movement and repetition—movement and repetition of the single signs constructing the compound are reduced. This applies most drastically to the first element of the compound.
2. Smoothing of transition between signs—movement used for transition between the two signs in a compound is reduced spatially and temporally. The two signs are signed closer to each other in signing space, the transition is faster, and the movement between them is smoother.

These morpho-phonological changes distinguish the compound from a phrase containing the two separate signs and shorten its duration to be more compatible with a duration of a simple lexical sign [69].

Therefore, compounds exhibit a case of syllables sequenced one after the other, and they are an example of a more sequential nature that can appear in sign languages, resembling the sequential nature of phonemes and syllables in spoken languages. Thus, a cognitive mechanism that handles phonological elements of signs must be able to deal with both simultaneous and sequential information. Note that a similar conclusion can be reached with regard to ordinary spoken phonology, if one decomposes phonemes into their constituent features, such as place of articulation and manner of articulation, voicing, aspiration, and frication, which have to be produced simultaneously.

This argues against a dedicated output buffer for signs with a radically different specialization from the one for spoken phonemes, even though, if the substrate for the two is indeed the same, it has to operate with different statistics in natural conditions—with words mostly being represented by several phonemes, or even several syllables, for many spoken natural languages; and, mostly by single signs composed of simultaneous features, for sign languages. It remains to be understood what the requirements of a system that can operate under both statistics are.

Moreover, sign-elements (handshape, movement, and location) most naturally correspond to the word’s phonemes. However, whereas well-formed signs must include specifications for all three elements, one for each element (in most morphologically simple signs), words vary regarding the number and types (consonants and vowels) of phonemes they comprise.

Therefore, in a most simplified scenario, in sign languages, the counterparts of spoken phonemes are sign-elements, and the features defining phonemes, and reflected in POB malfunction, such as place of articulation, manner, and voicing, are represented in sign language by features defining sign-elements. In information-theoretic terms, one may ask, given that the capacity of spoken and sign languages to convey bits of meaning per second appears similar, as corroborated by the practice of simultaneous translation, whether and in what sense one code may be more redundant or less error-prone than the other. The average production rate of syllables varies across spoken languages around 6/s, anti-correlated with the average entropy per syllable, to convey overall around 35–40 bits/s [72]. Thus, the POB is expected to churn out in the order of two three-syllabic words per second. Interestingly, about two per second is also an average observed production rate for sign languages [73]. The average sign “span” can also be measured and turns out to be, given considerable variability, 4–5 signs [74], perhaps lower but similar to the average span for three-syllabic words (which depends on the language, the kinds of stimuli selected, and on how it is measured), leading to the idea that a POB should normally operate correctly for at least of the order of 2.5 s, whatever the material it operates on.

## 3. Signing Output Buffer Function and Dysfunction

### 3.1. Working Memory Mechanisms in Signers of Sign Languages

Research on the pWM systems of deaf signers has revealed that the factors affecting these mechanisms in signers are very similar to their spoken language counterparts—the effects of word (or sign) length, phonological similarity, articulatory suppression, and irrelevant speech (or signing) were all shown to have an influence on pWM in both modalities [69,75,76,77].

A length effect of the signing pWM mechanisms was reported using a probe task—where ASL signers were requested to watch lists of signs containing either ’short’ or ’long’ signs, depending on the distance traveled in space within the sign–the length of the movement [78]. The participants were then presented with a sign that appeared on the list, and were requested to produce the sign that occurred immediately after the probe. It was found that the task was more difficult in lists of long signs—showing that a length effect exists in ASL signers, as found in speakers of spoken languages [21,22]. Note that this task does not involve recalling and producing the items in the list; thus, it mainly tests the phonological input buffer rather than the POB.

Mann [79] conducted a non-sign repetition task, in which deaf native or early signers of BSL were asked to repeat phonologically possible but non-existing BSL signs of different complexity. They showed that complex signs—containing complex (marked) handshape and complex (combination of path + internal) movement—were the hardest to repeat accurately, suggesting that phonological complexity of the sign might play a role in the pWM.

These findings show some major similarities between the phonological WM in deaf signers and hearing speakers, which suggests that the properties of the material per se, irrespective of modality, has a crucial role in shaping these mechanisms.

However, there is also evidence for differences between the phonological memory for signs and spoken words. One of them is a seemingly smaller span for lists of signs than for lists of words [80,81]. This difference was first explained by a longer articulatory duration for signs, but it was challenged by studies controlling the duration of signs relative to that of words [82]. However, comparing repetition of lists of letters between the two modalities, yielded no difference in span between fingerspelled letters (in signers) and auditorily-presented letters (in speakers) [83].

Geraci et al. [84] tested the sign span of deaf signers of Italian Sign Language (LIS) as compared to the word span of hearing speakers of Italian to the same signs translated to Italian. The articulation duration of the signs was significantly shorter than that of the words. The results showed that, even when articulation duration is controlled for, hearing participants had a greater span for Italian (written and spoken) words than deaf people for LIS signs. Nevertheless, the deaf signers outperformed the hearing speakers in a non-linguistic visuo-spatial WM task. It seems, then, that the capacity for signs in the pWM of deaf participants is smaller than the span for words in hearing participants; however, the use of a visuo-spatial language may enhance cognitive processes underlying central skills of sign language, leading to a better performance of the signers on the visuo-spatial span task.

To conclude, the research shows some major similarities between the phonological WM systems of signers and speakers in terms of effects on memory, with some potential differences, e.g., in span size.

### 3.2. Impairments to the POB acting on signs

Considering a smaller but comparable span between signs and words, similar effects reported for phonological WM mechanisms in signers and speakers, and some similarities in the phonological representations between the two modalities, one would expect that these mechanisms will also exhibit similar patterns in the cases of breakdown. [54,55,85] investigated the pattern of errors in native signers of Israeli Sign Language (ISL) who had Low Output Phonological Spans (Loops) in serial recall. The participants repeated lists of unrelated signs of different types, including simple lexical signs, compounds, and non-signs. The Loops made significantly more phonological errors than the control group in repetition of morphologically simple lexical signs within ISL sentences, and in repetition of morphologically simple non-signs. The phonological errors were mostly substitutions handshape (63%), but also of location (31%) and movement (7%). Many of these errors (54%) in the non-sign repetition test could be explained by migrations of sign-elements from neighboring signs—either from the same list or from the previous list. As discussed in Section 1, the functions of the POB include both storage of the phonological information and composition of the phonological units. In repetition of existing words/signs, the POB is supported by a long-term store, the POL, which stores the phonological information of familiar words/signs. However, in the repetition of non-words/non-signs the POB cannot be supported by the POL, and the phonological units stored in the POB are more susceptible to errors in storage and composition [86,87]. Thus, it is expected that the migration of units from neighboring words/signs would occur. [11] showed a very similar pattern of errors in reading pairs of words in a spoken language, Hebrew, where once the load exceeds the capacity of the POB, individuals make a large number of migrations of phonological units between neighboring words. A recent analysis of (spoken) non-word repetition data obtained by [88] in Hebrew speakers also revealed similar pattern of errors, with 65% of the errors explained by migrations within the word or from neighboring words (including transpositions, substitutions, and insertions of neighboring phonemes).

Haluts [54] also tested ISL signers in repetition and production of sentences containing function and number signs, and sign language unique morphologically complex structures, such as morphological facial expressions and classifier constructions. Morphological facial expressions are facial expressions that denote adjectives or adverbials, which occur together (simultaneously) with the sign and act as its modifiers. Classifiers are specific handshapes that denote groups of objects with some shared (physical or semantic) properties, which can be used together (simultaneously) with specific verbs to express movement or relative locations of objects. In repetition and production of sentences with these structures, the signers with low POB capacity made significantly more whole-unit errors, substituting one unit with another of the same category, just like the pattern seen in spoken languages: morphological affixes with other morphological affixes, function signs with other function signs, and number signs with other function signs. This was true despite the differences in the types of morphological affixes due to the different modality.

Therefore, the findings from POB-impaired signing and speaking individuals point to very similar functions performed by the POB in speaking and in signing, and suggest that the same, or very similar, mechanisms are responsible for processing of information in both modalities. Thus, it would be interesting to test whether the same network model can explain the processes formed by the POB in both spoken and sign languages, despite the possible differences discussed above regarding the more sequential or simultaneous nature of the phonological and morphological units in the different modalities.

In the current work, we explore how cortical mechanisms constrain the basic functions of the POB: receiving as input from a long-term store (the POL) phonological units of words and signs, and composing them correctly—sequentially or simultaneously. If we show that these different types of phonological units can be represented by a single network without mixing one type with the other, it would suggest that the same can be done with other types of units shown to lead to whole-unit errors within their category in POB impaired patients, e.g., morphological affixes, function words, and number words. We also aim to assess to what extent the model reproduces the same types of the phonological errors made by POB impaired signers and speakers.

## 4. A Potts Network Model of Cortically Distributed Compositional Memories

The Potts model offers a convenient mathematical framework to discuss the neural bases of language processes because of its key features:

- its units represent not neurons but small patches of cortex, in their tendency to approach one of *S* local dynamical states, so that attention is focused above the local circuitry and operations, which are assumed to be largely the same throughout.
- It assumes long-term memories of any kind to be distributed over many patches, even if localized at the gross system level, so that it can be analyzed statistically, adapting statistical physics techniques.
- In certain parameter regimes, it shows a tendency to generate spontaneous latching dynamics, or hopping among global cortical states, which can be utilized to model endogenous dynamics generated by cognitive processes.

In this section we review the basic features of the Potts model and in the next propose a first implementation of the phonological output buffer, which requires some additional features, and then discuss how POB-related data, in particular from Sign Language, may constrain the underlying neural computations.

### 4.1. The Potts Network With Discrete Long-Term Memories

One can adopt the perspective suggested by Valentino Braitenberg [89,90] by using Potts units, introduced in statistical physics in 1952 [91], to represent local cortical networks, that is, patches of real neurons, each patch taken to be endowed with its set of dynamical attractors, which span different directions in activity space. These states are identified with the states of the corresponding Potts unit, defined as pointing each along a different dimension of a simplex. Potts networks have been studied by [92,93,94,95], not as a model of the cortex, but rather as an interesting generalization of a Hopfield binary network [96], in which units are just binary variables, either active or quiescent. One can formally regard such a generalization still as an autoassociative network, but of *N* Potts units interacting through tensor connections. Long-term memories are stored in the weight matrix of the network and they are fixed, reflecting an earlier, possibly protracted learning phase [96]. Each memory μ is a vector or list of the states taken in the overall activity configuration by each unit *i*: $\xi_{i}^{\mu }$. We take each Potts unit to have *S* possible active states, labelled e.g., by the index *k*, as well as one quiescent state, k=0, when the unit does not participate in the activity configuration of the memory, which occurs with probability 1−a. Therefore, k=0,⋯,S, and each $\xi_{i}^{\mu }$ can take values in such abstract categorical set. The simil-Hebbian tensor weights read [92,97]

(1) $$c_{ij}J_{ij}^{kl}=\frac{c_{ij}}{c_{m}a\left(1-\frac{a}{S}\right)}\sum_{\mu =1}^{p}\left(\delta_{\xi_{i}^{\mu }k}-\frac{a}{S}\right)\left(\delta_{\xi_{j}^{\mu }l}-\frac{a}{S}\right)\left(1-\delta_{k0}\right)\left(1-\delta_{l0}\right)\;,$$

where i,j denote units, k,l denote states, *a* is the fraction of units active in each memory, $c_{ij}=1$ or 0 if unit *j* gives input or not to unit *i*, $c_{m}$ is the number of input connections per unit, and the δ’s are Kronecker symbols. The subtraction of the mean activity per state a/S ensures a higher storage capacity [92].

In a non-dynamical formulation, the units of the network are updated in the following way:

(2) $$\sigma_{i}^{k}=\frac{\exp\left(\beta r_{i}^{k}\right)}{\sum_{l=1}^{S}\exp\left(\beta r_{i}^{l}\right)+\exp\left[\beta \left(\theta_{i}^{0}+U_{i}\right)\right]}$$

and

(3) $$\sigma_{i}^{0}=\frac{\exp\left[\beta \left(\theta_{i}^{0}+U_{i}\right)\right]}{\sum_{l=1}^{S}\exp\left(\beta r_{i}^{l}\right)+\exp\left[\beta \left(\theta_{i}^{0}+U_{i}\right)\right]}\;,$$

where $r_{i}^{k}$ is the variable representing the input to unit *i* in state *k* within a time scale $\tau_{1}$ and $U_{i}$ is effectively a threshold. From Equations (2) and (3), we see that $\sum_{k=0}^{S}\sigma_{i}^{k}\equiv 1$, and also note that $\sigma_{i}^{k}$ takes continuous values in the (0,1) range for each *k*, whereas the memories, for simplicity, are assumed to be discrete, implying that perfect retrieval is approached when $\sigma_{i}^{k}≃1$ for $k=\xi_{i}^{\mu }$ and ≃0 otherwise.

If the connectivity matrix $c_{ij}$ is such that each Potts unit receives the influence of *C* other units, the quantities a,S and *C* (and the total number of units, *N*) are the main parameters that determine the storage capacity of the network. Global activity patterns, which are composed of local active and inactive states in the various units, can indeed be stored in the Potts network by the plasticity model in Equation (1). They are then (fledgling) attractor states, and the network functions as an auto-associative memory, retrieving one of *p* stored global activity patterns from a partial cue. Up to a limit $p_{c}$, which is roughly $p_{c}\approx CS^{2}/a$ —very large, given plausible assumptions about *C*, *S*, and *a*. Therefore, a model of long-term memory, which can hold millions of items in a network of the size of the human cortex—in stark contrast to the very limited capacity of short-term and working memory systems.

### 4.2. Potts Model Dynamics

When the Potts model is studied as a model of cortical dynamics, $U_{i}$ is written as $U+\theta_{i}^{0}$, where *U* is a common threshold acting on all units, and $\theta_{i}^{0}$ is the threshold component specific to unit *i*, but acting on all its active states, and varying in time with its own time course. This threshold is intended to describe local inhibitory effects, which in the cortex are relayed by at least three main classes of inhibitory interneurons [98] acting on ${\mathrm{GABA}}_{A}$ and ${\mathrm{GABA}}_{B}$ receptors, with widely different time courses, from very short to very long. To simplify the analysis, we write $\theta_{i}^{0}=\theta_{i}^{A}+\theta_{i}^{B}$ and assume the $\theta_{i}^{A}$ component to be very fast, and the $\theta_{i}^{B}$ very slow, both being driven up by recent activity in patch *i*. As discussed elsewhere [99], the dynamical behaviour of the Potts model is complex, different in distinct regions or phases of parameter space. It shows latching dynamics in a wider region, if both inhibitory fast and slow components are included. Therefore, we write, for the time evolution of the network

(4) $$\begin{array}{ccc} \tau_{1}\frac{dr_{i}^{k}\left(t\right)}{dt} & = & h_{i}^{k}\left(t\right)-\theta_{i}^{k}\left(t\right)-r_{i}^{k}\left(t\right)\\ \tau_{2}\frac{d\theta_{i}^{k}\left(t\right)}{dt} & = & \sigma_{i}^{k}\left(t\right)-\theta_{i}^{k}\left(t\right)\\ \tau_{A}\frac{d\theta_{i}^{A}\left(t\right)}{dt} & = & \gamma_{A}\sum_{k=1}^{S}\sigma_{i}^{k}\left(t\right)-\theta_{i}^{A}\left(t\right)\\ \tau_{B}\frac{d\theta_{i}^{B}\left(t\right)}{dt} & = & \left(1-\gamma_{A}\right)\sum_{k=1}^{S}\sigma_{i}^{k}\left(t\right)-\theta_{i}^{B}\left(t\right). \end{array}$$

The variable $\theta_{i}^{k}$ is a specific threshold for unit *i* in state *k*, varying with time constant $\tau_{2}$, and intended to model adaptation, i.e., synaptic or neural fatigue specific to the neurons active in state *k*. The field that the unit *i* in state *k* experiences is

(5) $$h_{i}^{k}=\sum_{j\neq i}^{N_{m}}\sum_{l=1}^{S}J_{ij}^{kl}\sigma_{j}^{l}+w\left(\sigma_{i}^{k}-\frac{1}{S}\sum_{l=1}^{S}\sigma_{i}^{l}\right).$$

Here, *w* is another parameter, the “local feedback term”, first introduced in [100], to model the stability of local attractors in the full model, as justified later with a semi-analytical derivation [97]. It helps the network converge towards an attractor, by giving more weight to the most active states, and, thus, it effectively deepens the attractors.

We assume, to be concrete, that $\tau_{A}\approx 10^{-3}s\ll \tau_{1}\approx 10^{-2}s\ll \tau_{2}\approx 10^{-1}s\ll \tau_{B}\approx 1s$. Note also that, in the limit $\tau_{2},\tau_{B}\to \infty$, the model would shed the adaptive character of its dynamics, and genuine attractor states would become indefinitely stable, and, hence, unsuitable for any spontaneous dynamics.

Stability would also be maintained if attractors were exceedingly “deep”, that is, if the local feedback *w* were strong enough to overcome the effects of the rising thresholds. Subsequently, the thresholds would stabilize at the asymptotic values $\theta_{i}^{k}\to {\bar{\sigma }}_{i}^{k}$ and $\theta_{i}^{B}\to \left(1-\gamma_{A}\right)\sum_{k}{\bar{\sigma }}_{i}^{k}$, and the input variables {r}’s would satisfy the asymptotic system $r_{i}^{k}=h_{i}^{k}-\theta_{i}^{k}$, where both the {h}’s and {θ}’s are functions of the $\left\{\bar{\sigma }\right\}$’s, hence of the {r}’s through Equations (2) and (3). Away from those limit cases, adaptation and slow inhibition tend to destabilize memory attractors. The one state that is guaranteed to remain stable, though, provided the constant threshold *U* takes a positive value, is the “global null state”, where all active states take low values, vanishing exponentially with β, and $\sigma_{i}^{0}≃1$ (the exact values depend on β and *U*).

Endowed with firing frequency adaptation and inhibition, the network displays, in certain conditions, latching dynamics [101], which is it hops from attractor to attractor, although the dynamics is often more complex, with the trajectory close to several attractors at the same time.

**Figure 5:**
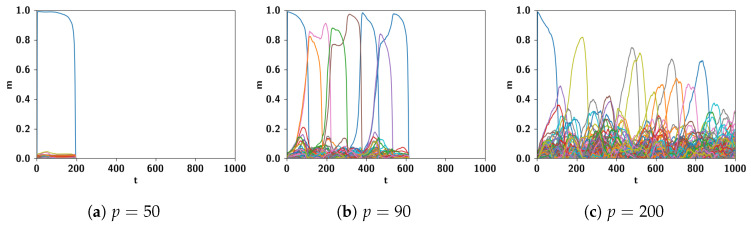
Examples of latching sequences. Three sequences with uncorrelated patterns of a network with only slow inhibition (*τ*_1_ = 3.33, *τ*_2_ = 100, and *τ_B_* = 10^6^). The *x*-axis corresponds to time, as measured by units of network updates. The *y*-axis measures correlations with different long-term memories, each in a distinct color. Increasing *p*, one observes different latching regimes. For too low *p*, in the no latching regime, there is only retrieval and the network cannot latch onto another pattern. Increasing *p*, one reaches the finite latching regime, middle, where one observes a finite sequence of well retrieved patterns. Increasing *p* further, right, in the infinite latching regime, sequences become indefinitely long, but, with increasingly large *p*, the network cannot retrieve any of them very well. Network parameters are *N* = 1000, *S* = 5, *a* = 0.25, *c_m_* = 150, *U* = 0.1, *β* = 11, *w* = 0.8.

Such dynamics is guided, absent other factors, by correlations between the different memory attractors [102]. Examples of latching sequences can be seen in Figure 5. Increasing the number of learned patterns, from p=50 to p=90 to p=200, the length of the sequence increases, but eventually to the detriment of the quality of retrieval. In [99], narrow bands are identified in p−S and p−C planes, where lengthy latching sequences co-exist together with good retrieval of each individual attractor visited by the network, both when inhibition is slow and when it is fast. Whichever parameter one considers, in fact, one finds that it can vary only in a narrow range between when latching is limited in duration and when its quality deteriorates. However, a recent result is that the combination of slow and fast inhibition expands the region in parameter space where protracted good quality latching prevails.

Because latching transitions occur with very uneven probability among different pairs of patterns, latching statistics defines which long-term memories are readily accessible from any given starting point, i.e., a sort of metric in memory space; or, more correctly, in the space of memory representations, which, if they for instance represent words, could be related to each other in their meaning, in word-form, phonetically, or having been associated in episodic memories, or in many other ways. Such types of correlations and the transitions they facilitate can be dissociated, once one introduces some internal structure in the so far undifferentiated, homogeneous Potts network.

#### Latching Guided by Heteroassociative Connections

The previous definitions are the foundations of a very simple model of the cortex. However, complex brain functions may need the introduction of rule-based memories (e.g., frequent associations, idioms, fixed sequences of actions, schemas) that cannot be simply described by a purely autoassociative network. Thus, we can consider the pairing of configuration μ to configuration ν, which is instructed to succeed it in time, μ→ν. These can be partial configurations, only defined over a specific subnetwork, and their heteroassociation may coexist with several other ones, μ→ν, μ→ρ, μ→ψ, ⋯. One might regard the long-term memory for a transition μ→ν, stored in a subnetwork of the cortex, as a schema, which favors its repetition, with different content in the complementary portion of the network that does not express the schema.

A conceptually distinct situation is when the pairing is only held in short-term memory, to remember a specific sequence for a short time. In this case, the favored transition μ→ν is taken to be unique, and reproducing it corresponds to successful remembering in the short term.

Patch-level implementation. Both these situation can be construed to involve the pairing of the complete or incomplete sets of adaptive thresholds $\left\{\theta_{i}^{k}\right\}$ that have been raised by the activation of configuration μ to the state variables $\left\{\sigma_{j}^{l}\right\}$ that have to be activated in configuration ν.

If μ is not a steady configuration of activity by an underlying extended cell assembly, in fact, but rather it represents a continuous attractor which at the microscopic, intrapatch level keeps changing in time, expressing the pairing in terms of the thresholds $\left\{\theta_{i}^{k}\right\}$ instead of the activity variables $\left\{\sigma_{i}^{k}\right\}$ implies that the transition is only favored once the continuous attractor has largely run its course, and it is close to being destabilized (by the very same $\left\{\theta_{i}^{k}\right\}$ thresholds).

Focusing for now only on the long-term heteroassociation, we can write the following expression for the couplings:

(6) $$J_{ij}^{kl,het}=\frac{c_{ij}\lambda }{c_{m}a\left(1-\frac{a}{S}\right)}\sum_{\mu =1}^{p}\sum_{\nu \neq \mu }^{p}G^{\mu \nu }\left(\delta_{\xi_{i}^{\mu }k}-\frac{a}{S}\right)\left(\delta_{\xi_{j}^{\nu }l}-\frac{a}{S}\right)\left(1-\delta_{k0}\right)\left(1-\delta_{l0}\right)$$

where λ modulates the strength of the heteroassociation and $G^{\mu \nu }=\left\{0,1\right\}$ defines the activity patterns associated one to the other. Plasticity is taken to have been refined over many repetitions of learning the rule, and so the coupling to be optimized for the long-term storage of these transitions. At this point, the new field *h* that unit *i* in state *k* experiences is

(7) $$h_{i}^{k}=\sum_{j\neq i}^{N}\sum_{l=1}^{S}\left[J_{ij}^{kl}\sigma_{j}^{l}+J_{ij}^{kl,het}\theta_{j}^{l}\right]+w\left(\sigma_{i}^{k}-\frac{1}{S}\sum_{l=1}^{S}\sigma_{i}^{l}\right).$$

This heteroassociative contribution will be further investigated in the next sections and will be developed according to the specific needs for modeling POB functions.

### 4.3. Short-Term Memory in the Latching Range Extended by Fast and Slow Inhibition

The Potts network that is recapitulated above is just a model of long-term memory. Can it be tweaked to serve also as a model of short-term or working memory? If so, it would demonstrate how memory operating on very different time scales may utilize the very same neural representations and the same associative mechanisms, based on plausible and unsupervised synaptic plasticity rules. Thus dispensing with the need to artificially create a separate short-term memory device. This is most relevant to the POB, since the POB is a WM system, which seems to also contain long-term mini-stores of pre-assembled units.

The core idea is that a few memory items, say, *M*, or also sequences of items, might be temporarily strengthened by modulating the value of some parameter, e.g., by increasing connection weights or lowering thresholds, to effectively bring the network across a phase transition, into a phase in which those items or those sequences are held effectively separate from the long-term ocean of all items and all possible sequences. The increase is assumed to be temporary, and once it subsides, the short-term memory has vanished. An important constraint is that, in keeping with the notion that the increase has a transient time course, we take the modified value of the parameter to be set very coarsely, in contrast with the parameters set to encode long-term memories, which in principle can be refined over many repetitions/recall instances, and can, therefore, be taken to be precisely set, even at the level of individual synaptic efficacies.

In light of this constraint, a limited latching range is a stumbling block: if the temporary modulation is set coarsely, the network is unlikely to follow a trajectory restricted to the items placed in short-term memory, or to latch at all. However, if the latching range is expanded, as it is seen to be in Potts networks endowed with the appropriate mix of slow and fast inhibition, a mechanism that is based on a coarse temporary modulation becomes cortically plausible, while also reproducing the similar prominence of slow and fast inhibition in the cortex [98].

One might consider different models, in which the temporary modulation affects different parameters. Here, we focus on those in which it acts on state-specific parameters. One can write, for example

(8) $$\tau_{2}\frac{d\theta_{i}^{k}\left(t\right)}{dt}=\sigma_{i}^{k}\left(t\right)-\theta_{i}^{k}\left(t\right)-\delta \theta \;H\left(\sum_{\mu =1}^{M}\delta_{\xi_{i}^{\mu },k}\right),$$

where *H* denotes the Heaviside function, and what the formula implies is that the modulation is applied indiscriminately to all states active in at least one of the *M* patterns; irrespective of whether those states are active in more than one of those patterns, or in other patterns. All of the Potts states that across units need to be in WM receive the same ’kick’ in the form of a lowered threshold. The kick δθ follows its own time course, and its coarseness requires that latching be effectively restricted to the *M* patterns, over a broad range of kick intensity. The probability that a given state in a unit receives the kick is close to Ma/S, hence as soon as M≃S/a it becomes impossible to distinguish between the *M* patterns in WM and all of the others in long-term memory. Therefore, the short-term or WM capacity is of order MS/a—a result that is confirmed by computer simulations.

Other models of temporary modulation are studied in detail elsewhere (manuscript in preparation).

## 5. A Structured POB Potts Network

Language production models typically consider the necessary processing stages that are needed to produce an utterance. In this sense, the phonological output buffer can be considered to be a special stage in which different streams of information have to converge to be transformed into a sequence of instructions to the articulatory system [103]. For our modeling purposes, we focus on a task involving only trisyllabic words, which the hypothetical speaker knows. This allows for us to consider only one source of input to the POB, namely the Phonological Output Lexicon (POL) [104,105]. For simplicity, we will consider the POL as a dictionary containing all of the relevant phonological information for uttering a word, and the POB as containing the repertoire of phonological units. These simplificatory assumptions are acceptable for an initial exploration, and they reduce the CPU time of our simulations by storing in the POL all the words that will be used to test the functioning of our POB model. We chose to refer to the phonological units of the spoken word as syllables, since three syllables are temporally analogous to the three sign-elements: as discussed in Section 2.4 above, both a single trisyllabic word and a single sign containing the three sign-elements take about 0.5 s to produce. However, from a linguistic point of view, and as discussed in Section 2 above, the formal correspondence would be between sign-elements and phonemes, which are the minimal contrastive elements in signs and words, respectively. In addition, the errors made by POB impaired patients are at the level of word phonemes and sign-elements, as explained in Section 3.2 above. Because we aim at this stage to show the possible representation of both sequential and simultaneous units by a network model of the POB, the sequential units that are represented by the model are not bound to a specific type, and later versions would have to account for more complex representations of units of different sizes. Nevertheless, the existence of sequential and simultaneous units will later allow us to compare the pattern of errors made by the model to the pattern of errors made by POB patients in spoken words with sequential units, and in signs with simultaneous units.

Our constraints on the form of the input are hopefully loose enough to be generalizable to other types of tasks and for other types of units (e.g., involving non-words, and analyzed also in terms of phonemes, or the other types of building blocks discussed above). The main function for this first attempt at implementing a neural POB stage is then to transform the compact package of phonological information coming from the POL into a temporal sequence of items. In future implementations of the model, further details and functions (e.g., phonotactic rules, morphological composition, production of affixes and function and number words/signs, morpho-phonological rules applied in the composition of stem and affix, etc.) may be added to the core mechanisms described here.

### 5.1. Network Structure

Our network model considers the interaction between the POL and the POB; therefore, we focus our attention on three main components: two autoassociative subnetworks, modeling the behavior of the POL and the POB, and the connections between the two networks, responsible for the transfer of information from the POL to the POB. No feedback connection from the POB to previous stages of the language production model is included for simplicity. Both subnetworks are modeled by Potts attractor neural networks. The POL receives its input in the form of an instantaneous cue to one of its stored patterns, namely the word to be uttered, from a previous stage and then transfers this information to the POB through heteroassociative connections, as described in Equation (6).

Parameter Setting

In all simulations, we have modeled the word buffer as a Potts network of $N^{POL}=600$ units, with $C_{m}^{POL}=90$ internal connections and $p^{POL}=200$ stored words. For both networks, the patterns stored were randomly generated without adding any sort of structured correlation, unlike in [102]. In order to prevent the buffer network from spontaneously latching, we chose the following set of parameters: $w^{POL}=0.45$, $\tau_{1}^{POL}=3.33$, $\tau_{2}^{POL}=33.3$, $\tau_{3}^{POL}=10^{6}$, S=7, a=0.25, β=12.5 and U=0.1. On the other hand for the POB we set the parameters to allow it to be driven into a latching regime when instructed by the POL. For the POB, we chose: $N^{POB}=200$, $C_{m}^{POB}=150$, $p^{POB}=200$, $w^{POB}=0.5$, $\tau_{1}^{POB}=3.33$, $\tau_{2}^{POL}=11.1$, $\tau_{3}^{POL}=10^{6}$, S=7, a=0.25, β=12.5, and U=0.1. The choice of a three times faster adaptation, determined by $\tau_{2}$, is motivated by the choice to focus on three-syllable words and, thus, to allow the POB to latch to all the three syllables while the word is still activated in the buffer. The number of heteroassociative connections between the two networks was fixed to $C_{m}^{het}=150$. This has to be interpreted as the number of units in the POL influencing each unit in the POB. In the following paragraphs, we illustrate the additional elements that define our model of the POB.

#### 5.1.1. Step 1: Cascade Input

In all simulations, we have only considered three syllable words. To instruct the POB on the correct sequence of three syllables composing a word, we worked on the shape of the heteroassociative matrix $G\in R^{p^{POL}x\;p^{POB}}$ in Equation (6). We assigned to each pattern stored in the word buffer a sequence of three syllables in a way that each syllable could be involved in composing only three words, each time in a different position. For instructing the sequential order of syllables for word W, we set $G^{W,S_{1}}=1.0$, $G^{W,S_{2}}=0.9$, and $G^{W,S_{3}}=0.8$, where $S_{1}$, $S_{2}$, and $S_{3}$ are the indices of the first, second, and third syllable of word W.

**Figure 6:**
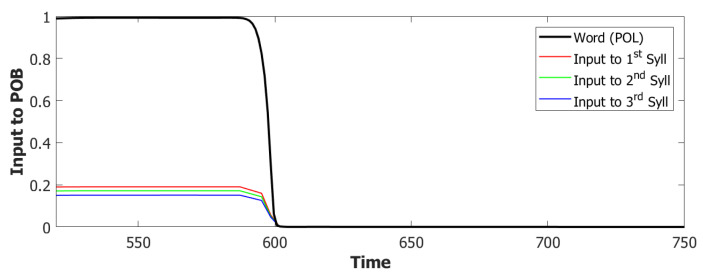
Input to each of the phonological output buffer (POB) syllables (coloured lines) associated to the active word in the POL (black line).

We used a value of λ=0.2 and a σ−σ interaction to implement the heteroassociation. This type of mechanism was preferred to the θ−σ one to favour a synchronous dynamic of the two subnetworks. As we can see from Figure 6, the POL sends a constant input with different strengths to the three syllables to be produced.

**Figure 7:**
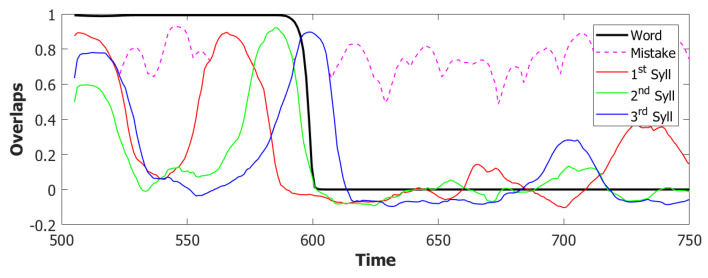
Example of the dynamics in the two subnetworks.

**Figure 8:**
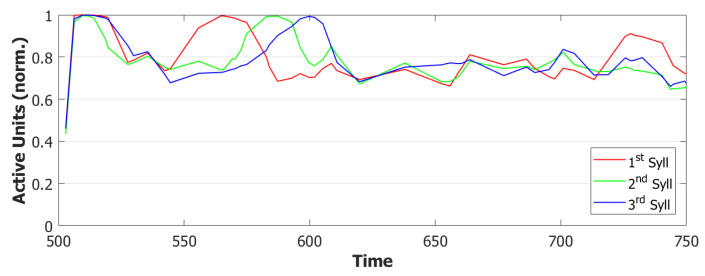
Normalized activity of the units encoding the three syllables. For each syllable, this was measured as $\left(1/aN^{POB}\right)\sum_{i}^{N^{POB}}\left(1-\sigma_{i}^{0}\right)\left(1-\delta_{\xi_{i}^{\mu }0}\right)$, where μ is the label of the syllable considered.

However the POB, even if sometimes it retrieves the correct sequence, as shown in Figure 7 by the red→green→blue color code, seems to enter a spontaneous latching phase where many wrong syllables are also retrieved. A possible origin of the problem can be seen by plotting the activity of the units encoding the 3 syllables (Figure 8). In an ideal scenario, the POB should be able to retrieve one syllable at a time, in the correct order and then turn off, waiting for the next utterance. What we observe here instead is sustained activity for all the units active in the three syllables, even after the end of the input.

#### 5.1.2. Step 2: Fast Inhibition

The constant signal from the POL induces an overactivation of the POB network, which is effectively driven in a spontaneous latching regime, often preventing it recovering the correct sequence. A possible solution would be to artificially transform the constant input from the word buffer into a sequence of instantaneous cues. However this approach would shift the problem of serializing the phonological information to the POL. Another option would instead be to reduce the activity of the network by increasing the effect of inhibition on its active units. One way to achieve this is to introduce a fast inhibition component, similar to the one that is considered in the previous Section.

**Figure 9:**
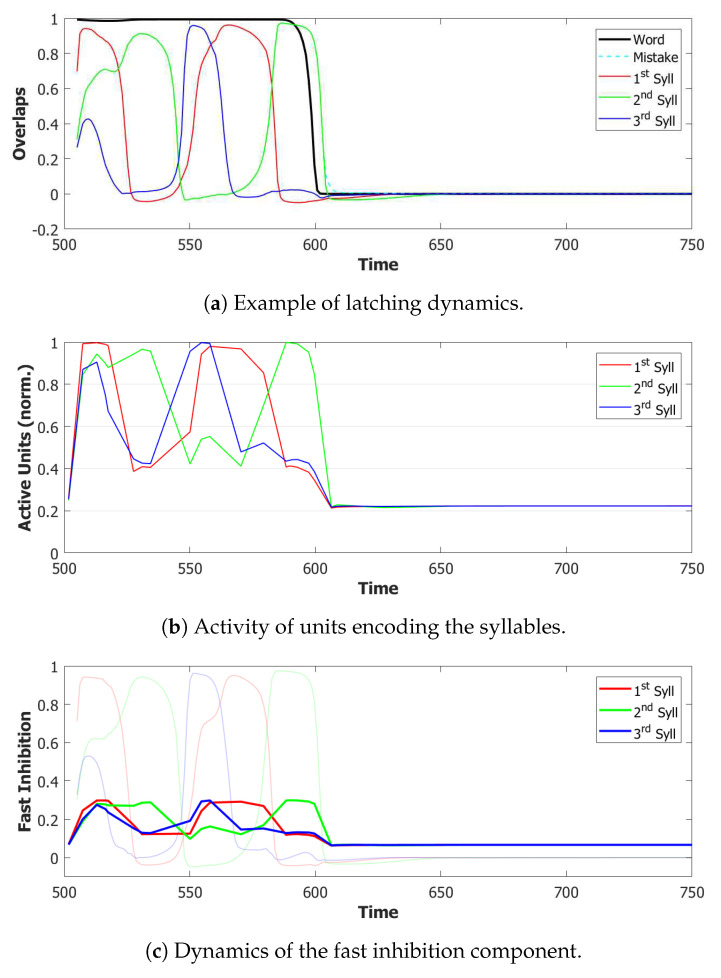
Example of a simulation with the introduction of a fast inhibition component. The latching dynamics now ends with the deactivation of the word in the buffer and it is mainly restricted to the correct subset of syllables.

For the simulations with this additional component, we used $\tau_{3}^{A}=2$ and a proportion of fast inhibition $\gamma_{A}=0.3$. As we can notice from the example in Figure 9a,b, the overactivation problem induced by the constant input is resolved by the introduction of this new component. With this configuration, the network, over three batches of 50 simulations, retrieved the correct sequence in the first three latching steps around 55% of the times. However the POB still “speaks” for more than requested, even if it does that without adding new and unrequested syllables.

**Figure 10:**
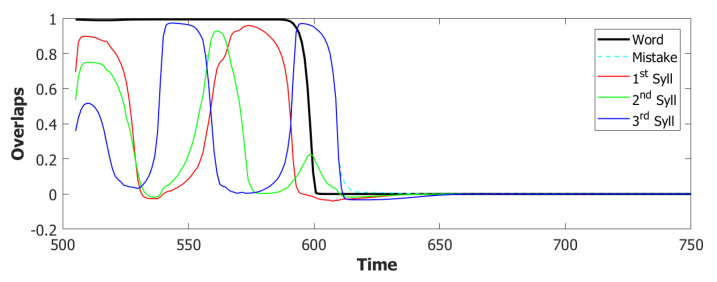
Example of a typical dynamical error induced by the initial co-activation rather than sequential activation of the three syllables. Note that the error is in the order of the syllables, in this instance also accompanied by the later mistaken activation of an extraneous pattern (dashed curve).

Coactivation of multiple syllables. A second and more subtle type of overactivation appears when comparing the three syllables with each other. Pairs of randomly correlated patterns share on average $a^{2}N$ active units. For our sparsity a=0.25, this roughly corresponds to a proportion of shared active units below 0.1. As we can see already in the example in Figure 9b, the minimum of this proportion of active units in our simulations fluctuates around a value of 0.4. This co-activation of syllables is indeed the main source of mistakes in this batch of simulations. Figure 9a and Figure 10 show the effect of the overactivation on latching for t<540, where all three syllables are simultaneously active in the network.

#### 5.1.3. Step 3: Dynamic Global Threshold

The simultaneous activation of the three syllables leads the network into a mixed state where multiple patterns are active together. When this situation occurs, the network has no immediately available pattern to latch to, leading to false starts and order errors of the kind shown in Figure 10.

The co-activation of multiple patterns can be interpreted as a lack of competition between the syllables, mainly driven by a weak constraint on the total number of simultaneously active units. To increase the selectivity of our network, we need to introduce a mechanism that penalizes units that are not aligned with the most active syllable. This new type of inhibition was introduced in our simulations as a dynamic component added to the previously defined constant global threshold *U*:

(9a) $$U\left(t\right)=U+\hat{U}\left(t\right)$$

(9b) $$\tau \;\frac{d\hat{U}}{dt}=\frac{1}{aN^{POB}}\sum_{i\in POB}\left(1-\sigma_{i}^{0}\right)-\hat{U}$$

where, for our simulations, we set the value of τ equal to $\tau_{3}^{A}$.

The introduction of the fast inhibition defined in Equations (9) can be justified as a rough first-order correction to the problem of discretizing the cortex into units when defining our Potts network. This approximation, however, should be acceptable only for small enough networks, where it is reasonable to assume that the local inhibition to a unit, being this a fictitious discretization of a continuous substrate, may influence also other units close by.

**Figure 11:**
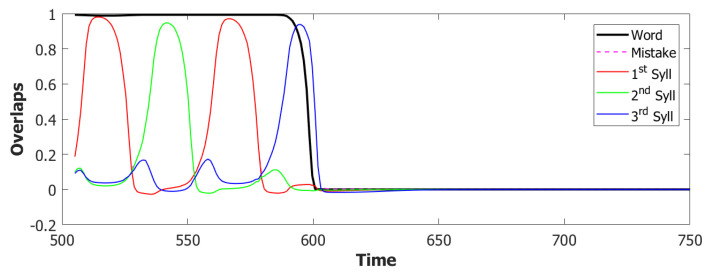
Example of latching dynamics after the introduction of a fast global inhibition component. The latching dynamics is more polished compared to previous simulations. Notice the suppression of extraneous syllables–the dashed pink curve barely appearing at the end.

Simulations that are performed with this new mechanism show a noticeable improvement in the quality of latching. Each syllabic utterance corresponds to an isolate latching step with no interference coming from other overlapping syllables. Nonetheless, the proportion of correct sequences decreased drastically in the way illustrated by the latching sequence in Figure 11, as will be discussed in depth in the next sections. The time dilation induced in the dynamics as a byproduct of the dynamic global inhibition introduced what we can define as a short-term memory issue in our simulations. A tentative solution will be addressed in the next, and final, step by modulating the adaptation component in the POB.

#### 5.1.4. Step 4: Slow Adaptation

**Figure 12:**
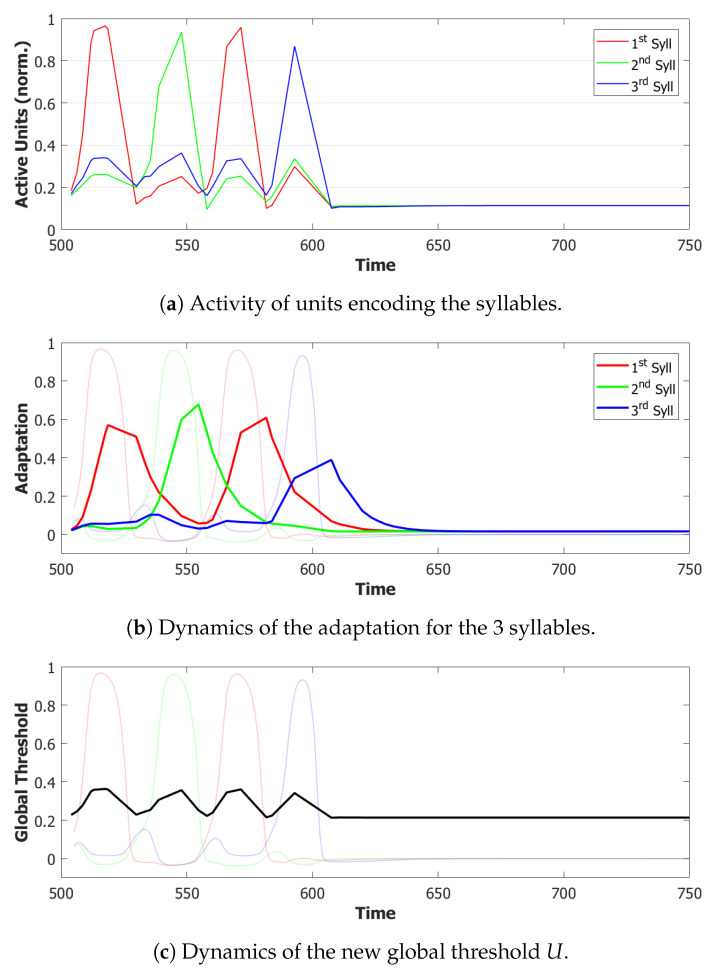
Dynamics of the main variables in the simulation in Figure 11. The effect of the new inhibition is to reduce the co-activation of multiple patterns.

Adaptation, modeled by the second equation in Equation (4), is the mechanism that forces active units to change their preferred state of activity once a certain amount has passed. Figure 12b shows the normalized amount of adaptation for each syllable. As we can notice from this example, at the time of the third utterance, the adaptation of the first syllable is already low enough to let it take advantage of its greater input and win the race against the third syllable. The time-constant $\tau_{2}$ is then the parameter that regulates the memory of the network about the previously active patterns. The choice of a shorter time-scale to allow for a faster dynamics in the POB also corresponds to faster forgetting of the previous states. To correct this behavior, without altering too much the dynamics, we introduced a second term of adaptation, similarly to what has been done to merge slow and fast inhibition in the third and fourth equation in (4).

(10a) $$\theta_{i}^{k}\left(t\right)=\theta_{i}^{k\left(slow\right)}\left(t\right)+\theta_{i}^{k\left(fast\right)}\left(t\right)$$

(10b) $$\tau_{2}^{\left(fast\right)}\frac{d\theta_{i}^{k\left(fast\right)}}{dt}=\gamma_{2}^{\left(fast\right)}\sigma_{i}^{k}-\theta_{i}^{k\left(fast\right)}$$

(10c) $$\tau_{2}^{\left(slow\right)}\frac{d\theta_{i}^{k\left(slow\right)}}{dt}=\left(1-\gamma_{2}^{\left(fast\right)}\right)\sigma_{i}^{k}-\theta_{i}^{k\left(slow\right)}$$

To limit the number of parameters, we set $\tau_{2}^{\left(fast\right)}=\tau_{2}^{POB}$ and $\tau_{2}^{\left(slow\right)}=\tau_{2}^{POL}$. For the proportion of slow and fast adaptation, we set $\gamma_{2}^{\left(fast\right)}=0.5$.

**Figure 13:**
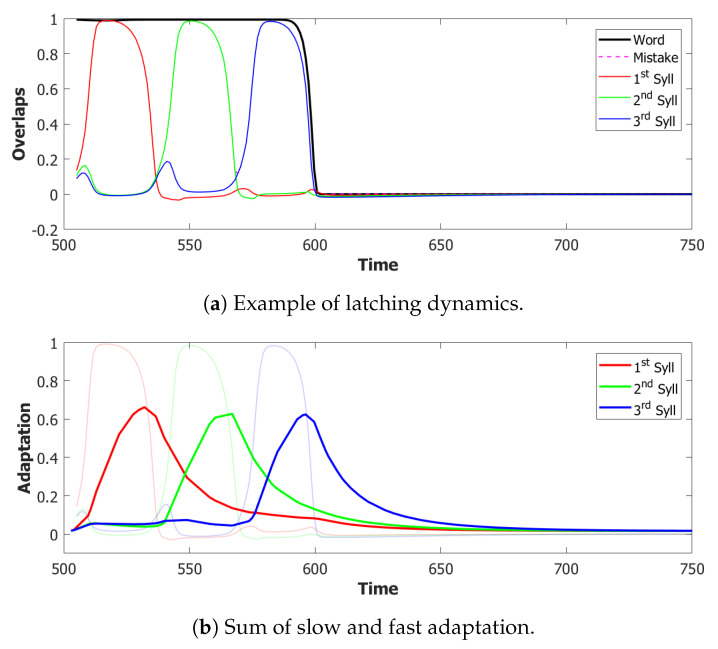
Example of a simulation with the introduction of a slow adaptation. The latching dynamics is more polished compared to previous simulations.

With this additional tool, many of the syllable repetitions were prevented (see Figure 13 for an example) and the performance of our model drastically improved to a value around 72% of correct sequences.

### 5.2. Simulation Results

#### 5.2.1. Performance of the POB Model and Points of Failure

In the previous section, we showed how we exploited the available mechanisms in our Potts neural network to build a basic model of the phonological output buffer. We run three batches of simulations, each having different sets of patterns for both the words and the syllables, to analyze the performance of the final model and where it breaks down. For each set, we stored in the network 50 word syllables associations that were all simulated by cueing the relative word in the POL. The resulting 150 simulations were then aggregated to analyze the performance of the network.

**Figure 14:**
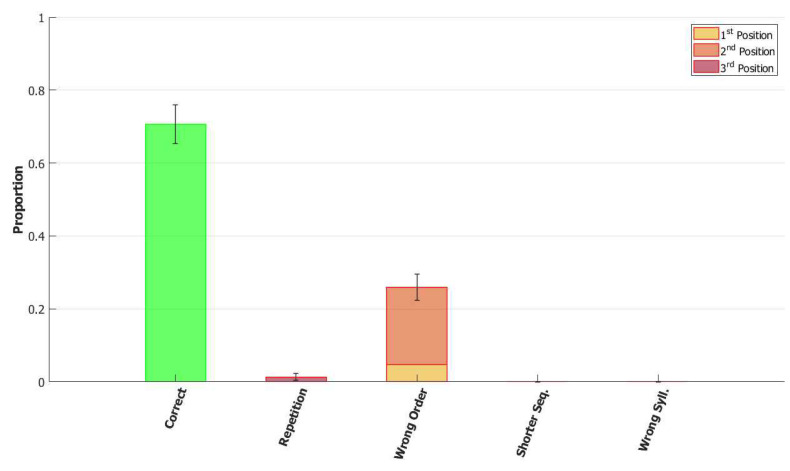
Distribution of errors in the first three utterances of the final POB model. Error bars represent the standard error of the mean. The intensity of the red shade codes for the position of the first wrong utterance, which one should note also in the following figures, is strongly constrained by considering only trisyllabic words.

In the (provisionally) final model, the majority of utterances were correct (72% accuracy), while the main type of errors committed by the network was to switch the position of two correct syllables, as shown in Figure 14. Transposition of phonological units, a type of migration, was found to be very frequent both in POB impaired signers and in POB impaired speakers, as discussed in Section 1 and Section 3.2, above. Interestingly, this type of error was also the most frequent error made by the model.

#### 5.2.2. Breaking the Network: Analysis of Errors

To better understand the role of the mechanisms included in the model we ran, with the same procedure of the section before, other rounds of simulation each time removing a single one of the added components and examining the type of errors that occur.

**Figure 15:**
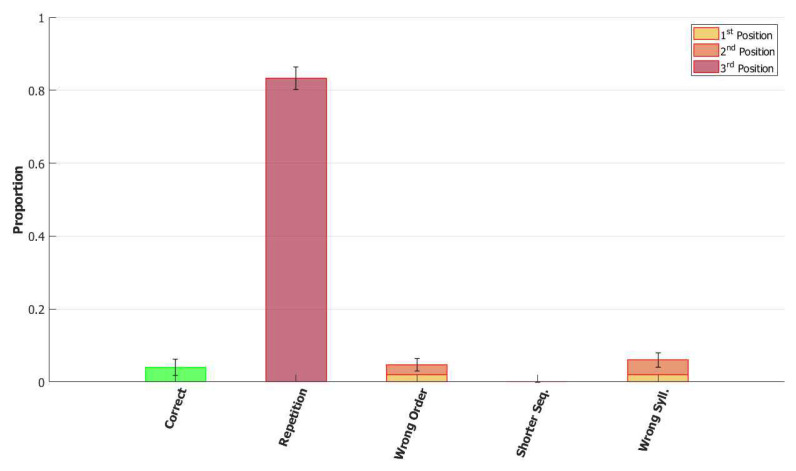
Distribution of errors in the first 3 utterances without slow adaptation, i.e., $\gamma_{2}^{\left(fast\right)}=1$, while all other parameters are the same as in the complete model. Color coding and error bars as in Figure 14.

**Figure 16:**
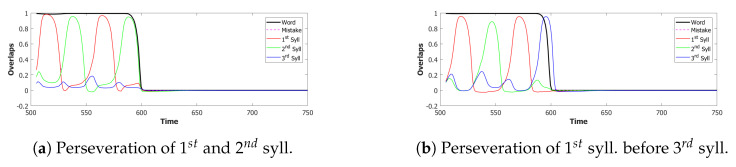
Examples of typical errors in a network with no slow adaptation. Both types of errors are classified as repetition errors.

*No slow adaptation.* Slow adaptation was introduced in the fourth step to help avoid triplets of syllables with repetitions of the kind $\left\{1^{st}\to 2^{nd}\to 1^{st}\right\}$. The analysis of the performance in Figure 15, once slow adaptation has been removed, indeed confirms the prevalence of these repetition errors. Examples of repetition errors are shown in Figure 16. The type of repetition in Figure 16b could also be listed as an addition error, with the intrusion of the first syllable. Repetitions and additions also occur with POB-impaired patients, and one may conjecture that these errors are normally suppressed by a control stage, operating with the POB, but with reduced effectiveness in these patients. Interestingly, our minimalistic model, with no control stage added, indicates that faulty syllable repetition may result also from a reduced slow adaptation component, thus suggesting a different point of failure, independent of external control stages.

**Figure 17:**
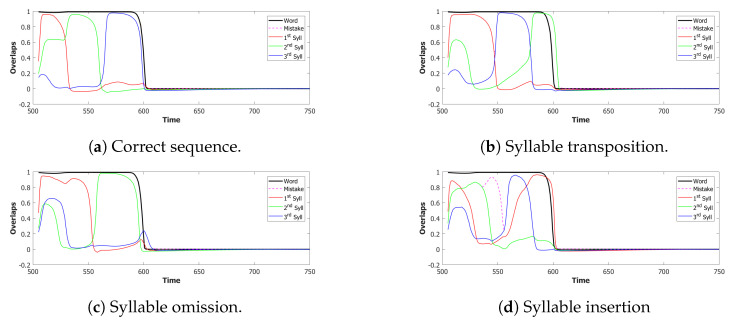
Examples of a correct sequence and of three errors in a network with no dynamic global inhibition.

**Figure 18:**
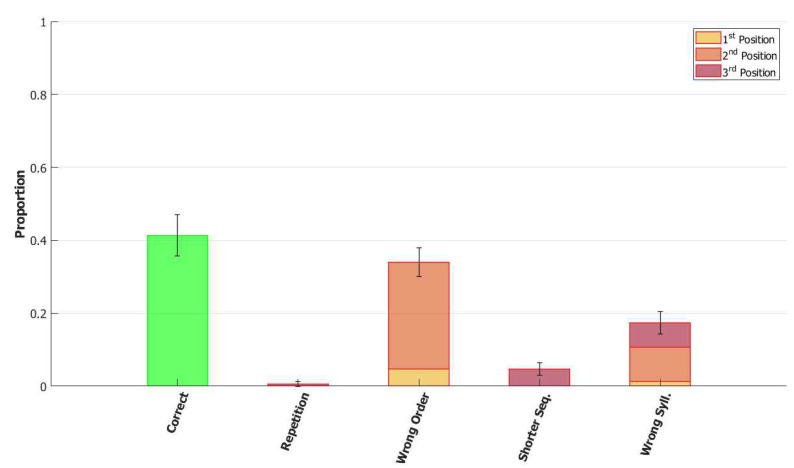
Distribution of errors in the first three utterances without dynamic threshold, i.e., U=0.216, with no temporal evolution for this parameter. Again, color coding and error bars as in Figure 14.

*No dynamic global threshold.* Simultaneous activation of multiple syllables lead us to the introduction of a competition mechanism in the model. In these simulations, we removed from the complete model the time evolving component of the threshold *U*. For a fair comparison, we assigned to *U* a higher value corresponding to the value assumed by Equation (9) for a network in the thermal state at a temperature $T=\frac{1}{\beta }$. In our case, we set U=0.216. This model showed a variety of types of errors and a low accuracy on correct sequences, as illustrated in Figure 17 and Figure 18. For simplicity, we categorized omissions in “Shorter Sequence” errors, independently of the omitted syllable, and syllable insertions in the “Wrong Syllable” class, to highlight the utterance of an intruded external syllable. All bisyllabic utterances only involved the correct syllables.

**Figure 19:**
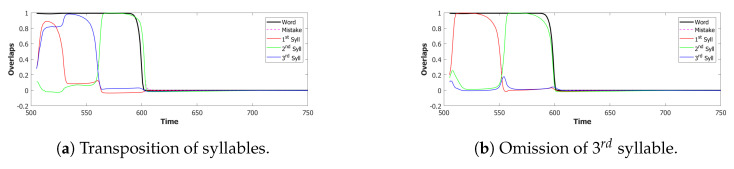
Examples of errors in a network with no fast local inhibition.

**Figure 20:**
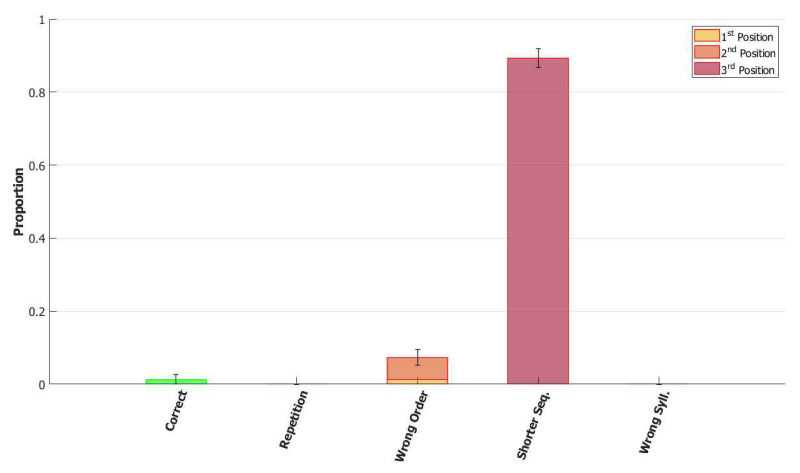
Distribution of errors in the first three utterances without fast local inhibition, i.e., $\gamma_{A}=0$, so that only slow local inhibition is present.

*No fast local inhibition.* Fast inhibition was the first “ingredient” added to the basic network. Simulations of the complete model, except for missing fast local inhibition, show the importance of this component, see Figure 19. Omission errors were the predominant type of error in these simulations. Very few (10%) trisyllabic words were indeed uttered by this network and almost never in the correct order, as shown in Figure 20.

With respect to the serial position effect, the model shows more errors for later serial positions, as often shown in studies of repetition. Interestingly, the argument was made [13] that this effect might support a different route, which does not use the POB, for single (existing) words, which is affected by a gradient in lexical activation, so that the activation of successive units in the lexicon decreases with position in the word. The notion is that if single words were also processed in the POB, conceived as a short term store, they should exhibit a U-shaped serial position curve. The Potts model offers a counterexample to this argument, showing that a network that can also operate as a short term store does not necessarily exhibit a U-shaped curve. Note, however, that the distinction between a downward gradient and a U-shaped curve is clearer with errors involving wrong syllables, insertions, or omissions, than with “wrong order” errors. Particularly so with trysillabic words, as used in our simulations, where “wrong order” involves either two or even all three of the syllables.

In summary, the type of errors made by the model when removing the different components correspond to similar errors made by POB impaired signers and speakers, as discussed above in Section 1 and Section 3.2. As mentioned above, “wrong order” errors correspond to transpositions of phonological units. “Repetition” corresponds to perseveration of units within and between words, “shorter sequence” corresponds to omission of units, and “wrong syllable” correspond to insertion of additional wrong units, all of which were reported to occur in individuals with impairments to the POB.

## 6. From POB to SOB

With respect to the buffer presumed to operate in sign languages, the capacity to acquire it and its homology with the spoken phoneme buffer suggest that they may share the same neural substrate, while usually operating on objects of different statistics. The underlying network mechanisms would then not be particular to the objects operated on, but would only reflect general entropic/statistical constraints and the neural wetware in which they are implemented. However, beyond the overall similarity, the efficiency of the spoken and signed code may be quantitatively different-–-it has been suggested that handshapes come much closer to a maximum entropy code than spoken phonemes, for example—and the required dynamics is qualitatively different—due to the largely simultaneous rather than sequential production of the constituents of a signed word.

Here, we sketch a first analysis of these constraints, using the very same Potts network model of cortical dynamics, a portion of which is taken to represent the objects held in the output buffer. To get to the essential difference between signs and spoken words, we focus on signs with three simultaneous elements and contrast them with three-syllable words, without implying, however, that the three main elements of a sign should be regarded as equivalent to syllables, rather than to phonemes or other conceptual units. We simply want to assess whether the cortical machinery that can, with all the pitfalls just reviewed, produce a sequence of three items in roughly half a second, can alternative produce three simultaneous items over the same time.

### 6.1. Signs Patterns

We now try to assess to what extent the same network can recall signs, through the parallel retrieval of their three constituent sign-elements (i.e., handshape, movement, and place of articulation). Thus, our first approach to a SOB model is to conceive a more suitable pattern structure for simultaneous activation. Therefore, the POB network developed before is subdivided into three clusters of Potts units, each assigned to an element of gesture. The corresponding Potts activity patterns are randomly generated on the relative clusters of N/3 units with sparsity $a^{SOB}$. However, this differentiation does not influence the syllable patterns for the spoken modality, which remain distributed over all the units with sparsity $a^{POB}$.

**Figure 21:**
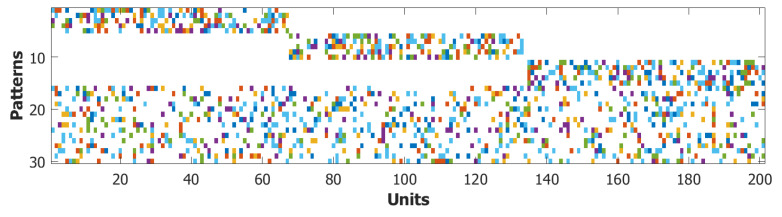
Distribution of activity patterns for a SOB–POB network with N=201 units. Differently colored cells represent active units in different states. White dots represent inactive units. Gesture elements, indexed from 1 to 15, are generated in three non-overlapping clusters of 67 units with a sparsity $a^{SOB}=31/67$ while syllable patterns, indexed from 16 to 30, are randomly distributed over all units with sparsity $a^{POB}=51/201$.

Signs and spoken words are then created by combining, with different statistics, patterns drawn from a pool of, respectively, 15 gesture elements, equally distributed between the three element types, and 15 syllables, generated, as shown in Figure 21. Each sign in our model is composed by one gesture element of each type for a total of 5×5×5 possible combinations. On the other hand, spoken words are composed by a sampling with no replacement procedure on the 15 syllables, with therefore 15×14×13 possible combinations. Note however that the substrate for the (model) sign constituents and the (model) spoken syllables is the same: the key point is the heterogeneity of the items represented by attractors of the same network; other items could then be numbers, function words, morphemes, etc.

### 6.2. Inhibition and Adaptation

The difference above, in the patterns that model the presumed cortical representation of words and signs, is significant enough to make, on its own, the POB model unable to operate with the new material. The dynamic global inhibition introduced in the POB model fulfilled, in fact, the requirement of competition between syllables. For the same buffer to operate as a SOB with simultaneous gesture elements, this competition mechanism is obviously detrimental. To allow the three elements to be simultaneously retrieved by the network, we need to separate what before was global, i.e., buffer-wide inhibition into a cluster-wide inhibition. In this new framework, the three clusters are subject each to its own inhibition. This *ad-hoc* tuning can be regarded as a refinement of the initial assumption of inhibition extending over the entire buffer, which itself was a refinement or first order correction to the tessellation of the cortex into discrete patches with inhibition only acting locally within each patch.

Adaptation is also a very important component in the proper operation of the buffer. The interaction between fast and slow adaptation allows to latch from one pattern to the next, by keeping in a form of short-term memory the previously uttered syllables. However, the SOB version of the model does not rely on a latching sequence of stored items, but just on their simultaneous and sustained activation. Therefore, in the Potts implementation signs do not require fast adaptation. To favor this behavior in a SOB–POB network, we modulate the value of the parameter $\gamma_{2}^{\left(fast\right)}$ introduced above, to account for the recruitment of a particular unit and state in sign-elements and in spoken syllables. In more detail, $\gamma_{2}^{\left(fast\right)}\left(i,k\right)$ was set to zero if unit *i* in state *k* was recruited only by sign-elements. In case both signs and syllables recruited unit *i* in state *k*, the value of $\gamma_{2}^{\left(fast\right)}\left(i,k\right)$ was calculated according to the following equation:

(11) $$\gamma_{2}^{\left(fast\right)}\left(i,k\right)=0.5\frac{n_{syll}}{n_{sign}+n_{syll}},$$

where $n_{syll}$ and $n_{sign}$ are the number of syllables and sign-elements with $\xi_{i}=k$. In all other cases, the value of $\gamma_{2}^{\left(fast\right)}\left(i,k\right)$ was set to 0.5.

**Figure 22:**
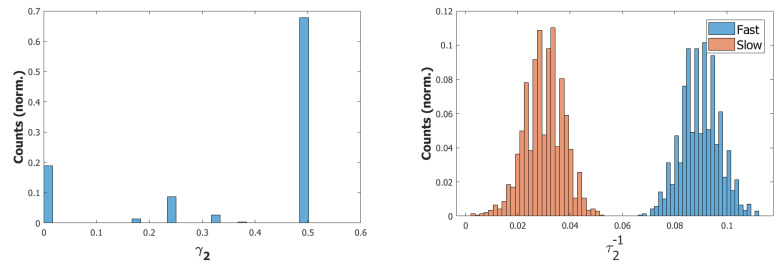
Example distributions of $\gamma_{2}^{\left(fast\right)}$ and ${\left[\tau_{2}\right]}^{-1}$.

As a final ingredient, with the purpose of generalizing as much as possible the SOB-POB model, we modify the previously constant adaptive timescales $\tau_{2}^{\left(fast\right)}$ and $\tau_{2}^{\left(slow\right)}$ into random variables. Thus, for each unit *i* in state *k*, we set the fast- and slowly-adaptive timescales as:

(12a) $${\left[\tau_{2}^{\left(fast\right)}\left(i,k\right)\right]}^{-1}=N\left(b^{fast},\frac{b^{slow}}{4}\right)$$

(12b) $${\left[\tau_{2}^{\left(slow\right)}\left(i,k\right)\right]}^{-1}=N\left(b^{slow},\frac{b^{slow}}{4}\right)$$

where $b^{fast}=0.09$ and $b^{slow}=0.03$ correspond to the inverse of the previously constant timescales $\tau_{2}^{\left(fast\right)}$ and $\tau_{2}^{\left(slow\right)}$, as shown in Figure 22.

### 6.3. Simulating the SOB–POB

For the simulations, we stored 25 signs and 25 words in the connections between the Output Lexicon and the SOB-POB network. Signs and words were generated from the sets of 15 gesture elements, being equally divided among the handshape, movement, and place of articulation categories, and 15 syllables. Network parameters were mostly kept as in previous POB simulations. For the Phonological Output Lexicon (we keep this name even though now it is taken to store also signs), we used $N^{POL}=600$, $C_{m}^{POL}=90$, $p^{POL}=200$, $w^{POL}=0.45$, $\tau_{1}^{POL}=3.33$, $\tau_{2}^{POL}=33.3$, $\tau_{3}^{POL}=10^{6}$, a=0.25, S=7, β=12.5, and U=0.1. For the buffer we chose instead: N=201, $C_{m}=150$, w=0.5, $\tau_{1}=3.33$, $\tau_{2}^{\left(fast\right)}=11.1$, $\tau_{2}^{\left(slow\right)}=33.3$, $\tau_{3}^{A}=2$, $\tau_{3}^{B}=10^{6}$, S=7, β=12.5, U=0.1, $a_{word}=51/N$, $a_{sign}=31/N$, and p=200 patterns, of which only 30 were used to generate signs and words.

The number of heteroassociative connections between the two networks was fixed to $C_{m}^{het}=150$. The simultaneous activation of sign-elements does not require a cascaded input to the buffer; therefore, we set to 1 the entries in the heteroasociative matrix *G* for each of the three elements associated to a sign. However, initial simulations showed a need for a stronger input to obtain the full activation of all sign-elements. For this reason, we set a higher value of λ for signs as compared to words. While, for words, we kept $\lambda_{word}=0.2$, for signs we used $\lambda_{sign}=1.4$.

### 6.4. Signs and Words: Results

The goal of the SOB–POB model is to assess the capability of the Potts attractor neural network to retrieve the correct components of signs and words, without mixing the two classes of materials stored in its connections. Note that what we use here are, again, highly simplified models of real ’signs’ and ’words’, differing only in the simultaneity of their respective components, and in an artificially clearcut manner; so, any inference about the mixing of other classes of material would at best be very indirect.

In the first set of simulations we have studied the behaviour of the network when cued with short sequences of signs and words, concurrently stored in the same network.

**Figure 23:**
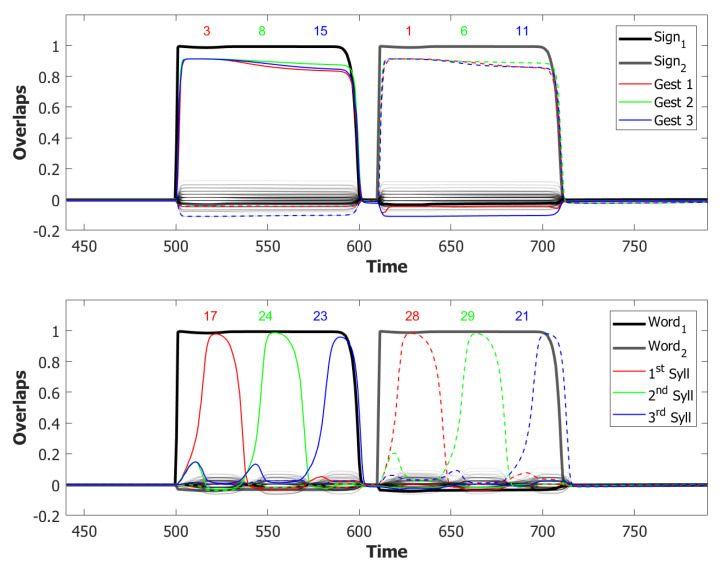
Example of two sequences of signs and words. Colored lines represent the overlaps of correct items for the first (full lines) and second (dashed lines) recalled patterns. Colored indexes show the pattern index for the correct stored item. Both sequences are perfectly retrieved.

**Figure 24:**
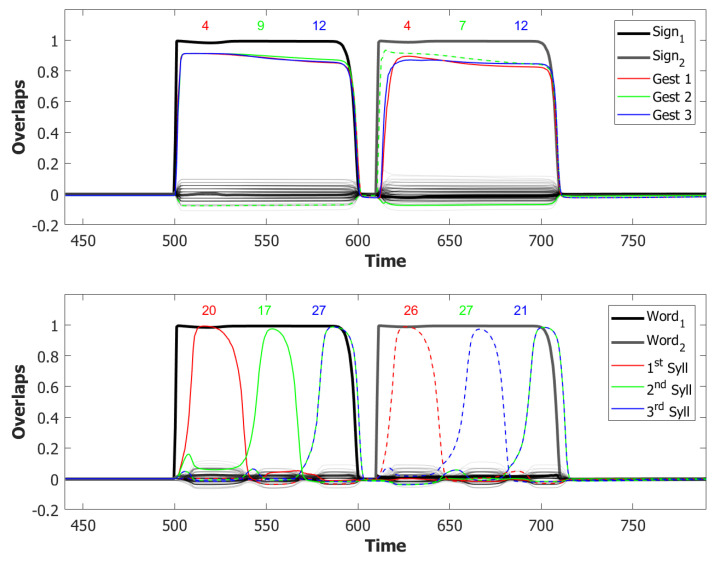
Example of two sequences of signs and words with repeated gestures and syllables. Colored lines represent the overlaps of correct items for the first (full lines) and second (dashed lines) recalled patterns. Colors indicate the pattern index for the correct stored item. The sign sequence is correctly retrieved, with only a short delay in the activation of the repeated syllables. Conversely, the syllable sequence shows an inversion of the second and third syllable in the second word, possibly due to the immediate repetition of syllable 27 from the first word.

Figure 23 shows two examples of successful sequence recall, one of two signs and one of two words. Simulations of our generalized model of an output buffer show that our Potts network succeeds in retrieving the correct patterns in the two implemented modalities. Interestingly, the network in this regime has higher accuracy with signs, even when the two cued patterns share one or more gesture elements. Indeed, we have not found any error in sign retrieval, but only a slight delay in the activation of previously activated gestures. On the other hand, the consecutive cueing of words is more prone to syllable inversions, as shown in Figure 24. This result suggests a more complex processing for sequential recall rather than simple memory retrieval in our model.

**Figure 25:**
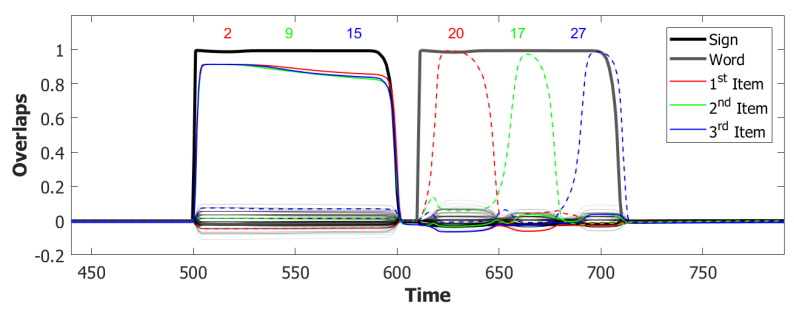
Example sequence of a sign followed by a word. Full colored lines represent the overlaps for correct gestures while dashed colored lines represent correct syllables. Colors indicate the pattern index for the correct stored item. In this example, both sign and word are perfectly retrieved.

A second relevant result, which might well apply to mini-stores in general, e.g., to those presumed to hold number words, function words, and morphological affixes, is the functional separation of contents in the same physical buffer. The different compositions of words and signs prevents in fact the network from mixing gestures and syllables, even when rapidly cueing signs and words one after the other, as shown in Figure 25.

Altogether our simulations suggest that a joint SOB–POB network might be able to recall the correct items, despite the encoding of mixed materials, namely sign-elements and syllables, encoded with different statistics.

**Figure 26:**
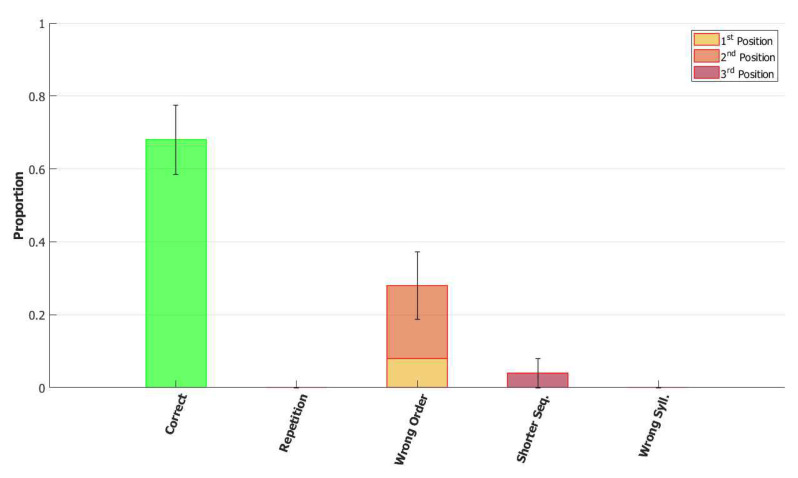
Performance of the SOB-POB network in retrieving the correct sequence of syllables. The percent of correct sequences is similar to that in a purely phonological network. No pattern belonging to the sign class is retrieved when recalling a word, as shown by the Wrong Syllable bar.

To confirm this conclusion, we checked the performance of the model in a second set of simulations, by cueing the network once for each stored word, when both types of materials have been included in the learning process. Figure 26 shows the statistics of the errors committed by (the POB component of) the SOB–POB network. The proportion of correct retrieval, order errors, and length errors remains similar to that of the purely ’phonological’ model in the previous section. Furthermore, no gesture intrusion appears in word retrieval, indicating the separation of materials in our model.

## 7. Discussion

Simulations of the (spoken) POB model indicate a reasonable performance in producing trisyllabic utterances, and where it is more prone to break down. The core functionality that this network simulates is to disentangle the compact information on the full (morphologically simple) word, stored in the POL, by transforming it into an ordered sequence of phonological units. In the Potts network, this occurs via purely associative mechanisms and a suitable mix of inhibition and adaptation, with no engineering and no language-specificity. We have shown that it can produce correctly utterances of three sequential (or simultaneous) units, which are stored in a long term store—the POL. When the produced utterance is incorrect, the model presents a similar pattern of errors to the one produced by POB patients. While the role of the POB in human speech production is of course more complex than the one assessed here, involving phonotactic and content-dependent rules (e.g., those pertaining to number words, to clipping and relaxed pronunciation, etc.), this first-order description already captures interesting features of human performance.

Disrupting basic neural mechanisms in the model produces error patterns that may be compared with individuals with specific POB impairments. Interestingly, each of the mechanisms, namely adaptation, local, and global inhibition, once removed produces a different pattern of errors in the utterances, as shown in Figure 15, Figure 18, and Figure 20. It should be noted that the current implementation does not consider feedback connections from the POB to the POL, which would be able to correct some of the wrong outputs reported in Figure 14 by modulating the strength of the inputs to the POB; still, there was broad similarity between the types of errors the model made and the ones POB patients make in spoken (and sign) languages. A future version of the model could include such connections, possibly increasing the accuracy of the utterances and, together with an additional ability to handle input that does not exist in the lexicon, it should be able to correctly reflect the difference between retaining words and non-words. Already with this implementation, however, the encouraging results suggest the suitability of the Potts network for modeling not only higher cognitive functions, but also specific low-level computations.

Finally, the last section demonstrates how, with limited changes in the distribution assumed for the underlying neural patterns of activity, the same network can act as a buffer for signs, with no mixing between sign-elements and spoken word syllables. Research has shown that individuals with POB impairments make whole-unit errors within categories: morphological affixes are substituted with other morphological affixes, function words with function words, and number words with number words—which supports the existence of different long-term mini-stores of these different types of units.

The model was able to produce both simultaneous and sequential utterances, without mixing them. The distinction made by the model between phonological units of signs and words suggests that the functional segregation of the different types of units in the output buffer may not reflect anatomical segregation of the relevant neural populations, but solely a difference in the statistical distribution of the corresponding neural activity. This, in a nutshell, is the change in perspective afforded by moving from a connectionist model (where either the coding is local, e.g., a phoneme unit is coded by a specific neuron or small group of neurons, an anatomical unit; or, it is distributed, but there is no dynamical “unit” to realize the discrete nature of the phoneme) to an attractor model, in our case the Potts network (where the attractor state is a dynamical unit with its discreteness, suitable to represent a phoneme, and it can be distributed on the same anatomical region as many other attractor states, while maintaining functional segregation). This ability sets the foundations for a future model to understand how different types of stimuli (e.g., function and number words, morphological affixes), which were shown to be prone to whole-unit within-category errors in POB patients, can be handled by the model without making between-category errors and without e.g., breaking a number word into its constituents. The non-implication of anatomical by the observed functional segregation might thus facilitate the further developments of theories of language processing, and of memory in general.
